# Pessary or cerclage (PC study) to prevent recurrent preterm birth: a non-inferiority, randomised controlled trial

**DOI:** 10.1016/j.eclinm.2024.102945

**Published:** 2024-11-25

**Authors:** Annabelle L. van Gils, Charlotte E. van Dijk, Bouchra Koullali, Malou A. Lugthart, Bo B. Bet, Maud D. van Zijl, Marijke C. van der Weide, H. Marieke Knol, Begoña Martinez de Tejada, Sanne J. Gordijn, Eline S.A. van den Akker, Marieke Sueters, Marjon A. de Boer, Brenda B.J. Hermsen, Yolanda M. de Mooij, Sabina de Weerd, Wilhelmina M. van Baal, Marion E. van Hoorn, Martijn A. Oudijk, Brenda M. Kazemier, Ben Willem J. Mol, Eva Pajkrt, PC Research Group, Eva Pajkrt, Ben Willem Mol, Brenda Miranda Kazemier, Maud Désirée Van Zijl, Bouchra Koullali, Malou Anne Lugthart, Bo Belle Bet, Charlotte Elisabeth Van Dijk, Annabelle Liselotte Van Gils

**Affiliations:** a Department of Obstetrics and Gynaecology, Amsterdam UMC Location University of Amsterdam, Meibergdreef 9, Amsterdam, the Netherlands; b Amsterdam Reproduction and Development Research Institute, Amsterdam, the Netherlands; c Department of Obstetrics and Gynaecology, Isala Hospital, Zwolle, the Netherlands; d Department of Paediatrics, Gynaecology and Obstetrics, University Hospitals of Geneva and Faculty of Medicine, Geneva, Switzerland; e Department of Obstetrics, University Medical Centre Groningen, University of Groningen, Groningen, the Netherlands; f Department of Obstetrics and Gynaecology, Onze Lieve Vrouwe Gasthuis, Amsterdam, the Netherlands; g Department of Obstetrics and Gynaecology, Leiden University Medical Center (LUMC), Leiden, the Netherlands; h Department of Obstetrics and Gynaecology, Amsterdam UMC, Location Vrije Universiteit Amsterdam, Boelelaan 1117, Amsterdam, the Netherlands; i Department of Obstetrics and Gynaecology, Zaans Medisch Centrum, Zaandam, the Netherlands; j Department of Obstetrics and Gynaecology, Albert Schweitzer Hospital, Dordrecht, the Netherlands; k Department of Obstetrics and Gynaecology, Flevoziekenhuis, Almere, the Netherlands; l Department of Obstetrics and Gynaecology, HAGA Hospital, The Hague, the Netherlands; m Department of Obstetrics and Gynaecology, Monash University, Clayton, Australia; n Aberdeen Centre for Women's Health Research, School of Medicine, Medical Sciences and Nutrition, University of Aberdeen, Aberdeen, UK

**Keywords:** Preterm birth, Recurrence, Pessary, Cerclage, Cervical insufficiency

## Abstract

**Background:**

Previous spontaneous preterm birth (sPTB) is a strong risk indicator for recurrent preterm birth (PTB). Cervical cerclage is an accepted intervention to prevent recurrent PTB in high risk patients. Cervical pessary might be a less invasive alternative. The objective of this study is to determine whether a cervical pessary is non-inferior to cerclage in the prevention of recurrent PTB.

**Methods:**

We performed an international, open-label, non-inferiority, randomised controlled trial in 21 hospitals between March 2014 and December 2022. We included singleton pregnancies with an indication for cerclage based on either multiple previous sPTBs <34 weeks or with a previous sPTB <34 weeks and an asymptomatic mid-trimester short cervix (≤25 mm). Randomisation was 1:1, stratified by centre and indication, to cervical pessary or vaginal cerclage. Primary outcome was PTB <32 weeks. Secondary outcomes included (s)PTB rates, obstetric, and maternal outcomes and a composite of adverse perinatal outcomes including perinatal mortality and severe neonatal morbidity. Analysis was by intention-to-treat. Treatment effect was expressed as relative risk (RR), absolute risk difference (aRD) and 95% confidence intervals (CI). Sample size was calculated at 400 participants with a non-inferiority margin for pessary of 10%, meaning that non-inferiority is proven if the upper limit of the CI of the risk difference is <10%. Trial registration at ICTRP: NL-OMON26958.

**Findings:**

We randomised 261 participants to pessary (n = 133) or cerclage (n = 128). After the third interim analysis (n = 228 participants), recruitment was halted due to safety concerns and the apparent challenge in establishing non-inferiority of pessary treatment. PTB <32 weeks occurred in 44/130 cases after pessary vs 30/125 cases after cerclage (33.8% vs 24.0% aRR 1.4, 95% CI 0.95–2.1, p = 0.09, aRD 9.8% 95% CI −1.2 to 20.9). The composite of adverse perinatal outcomes occurred in 42 cases after pessary compared to 29 cases in cerclage (32.2% vs 23.2%; RR 1.4 95% CI 0.93–2.1 p = 0.1) and consisted mainly of perinatal death (22.3% vs 14.4% RR 1.5 95% CI 0.9–2.6 p = 0.1).

**Interpretation:**

Non-inferiority of cervical pessary compared to cerclage in preventing recurrent PTB <32 weeks was not proven. Cerclage is the recommended treatment.

**Funding:**

ZonMw (#837002406), a Dutch Organisation for Health Research and Development.

**Research in context**

**Evidence before this study**

Cervical cerclage is an effective intervention to prevent recurrent PTB and overall perinatal morbidity and mortality in high risk patients, such as individuals with a history of multiple previous sPTBs or with previous sPTB and an asymptomatic short cervix <25 mm. While suggested as an alternative for cerclage, the use of a cervical pessary to prevent recurrent PTB in these subgroups is not yet supported by current evidence. Recently published systematic reviews stated that additional randomised trials are necessary to determine optimal management.

**Added value of this study**

This randomised study, comparing the effectiveness of cervical pessary compared to cervical cerclage, shows that non-inferiority of pessary treatment compared to cerclage could not be proven. A cervical pessary should not be used to replace cervical cerclage in high risk individuals with previous sPTB.

**Implications of all the available evidence**

In individuals with an indication for an intervention based on multiple previous sPTBs <34 weeks or a combination of previous sPTB and a short cervix, cerclage is the recommended treatment.

## Introduction

Preterm birth (PTB) poses substantial risks to neonatal health and remains a major global concern.^1^ Previous spontaneous preterm birth (sPTB) is a strong risk indicator for recurrent preterm birth (PTB), making it a prime target for prevention efforts.^2^^,^^3^ This recurrence risk is highest when previous sPTB occurred early in pregnancy and is estimated to be 30% following previous sPTB <37 weeks.^3^

For individuals with a history of multiple preterm deliveries due to suspected cervical insufficiency, early mechanical intervention with cerclage treatment is indicated before 16 weeks of gestational.^4^^,^^5^ In other cases of previous sPTB <34 weeks, serial measurements of cervical length (CL) are advised. If an asymptomatic short cervix ≤25 mm is detected before 24 weeks of gestation, the risk of recurrent PTB is further increased and a cervical cerclage is indicated.^5^, ^6^, ^7^

While a cerclage is used by convention, cervical pessary is suggested as a less invasive alternative. Previous trials found promising results with regards to the effectiveness of cervical pessary in the prevention of (recurrent) PTB.^8^^,^^9^ As direct comparisons of cervical pessary to cerclage are lacking, randomised trials to compare both interventions are needed.^7^^,^^10^^,^^11^

We aimed to evaluate whether a cervical pessary is non-inferior to cervical cerclage in the prevention of recurrent PTB in individuals with a singleton pregnancy and an indication for a cerclage.

## Methods

### Study design and oversight

We performed a multinational, open-label, non-inferiority, multicentre randomised controlled trial (RCT) in nineteen hospitals within the Dutch Consortium for Healthcare Evaluation and Research in Obstetrics and Gynaecology (NVOG Consortium), and two additional sites located in Italy and Switzerland. The study protocol has been published previously.^12^

### Participants

Individuals with a singleton pregnancy were eligible if they had at least one previous sPTB <34 weeks of gestation and had an indication for a cerclage based on either a history of multiple previous PTBs <34 weeks or asymptomatic short CL ≤ 25 mm on ultrasound before 24 weeks of gestation. Individuals <18 years, not able to give informed consent, or with the following medical conditions visible at time of randomisation were not eligible: placenta praevia, vasa praevia, premature pre-labour rupture of the membranes (PPROM), cervical dilatation ≥3 cm, CL < 2 mm, identified major congenital abnormalities, and clinical signs of intra uterine infection.

### Randomisation and masking

After written informed consent, participants were randomly allocated to receive a cervical pessary or a cervical cerclage, in a 1:1 ratio stratified per centre and per indication with a variable block size of 2 and 4. Randomization was centrally controlled using an online computerized randomisation service (ALEA Clinical software version 16, FormsVision, Abcoude, The Netherlands) and from July 2022 in Castor (Electronic Data Capture v2022.3.2.0). Blinding was not possible due to the nature of the interventions.

### Procedures

CL measurement was carried out from 14 weeks of gestation by transvaginal ultrasonography with a 5-MHz transducer, but was not necessary in those considered for an obstetric history indicated intervention. CL was measured according to the criteria of the Society for Maternal and Fetal Medicine.^13^ The presence or absence of funneling at the internal os was recorded. Research nurses and health care providers trained according to Good Clinical Practice guidelines (GCP) informed eligible individuals, obtained informed consent, performed randomization, and collected data.

An Arabin pessary size small (65/25/32 mm), medium (70/25/32 mm) or large (70/25/35 mm) (CE0482, MED/CERT ISO 9003/EN 46003; Dr Arabin GmbH and Company, KG; Witten, Germany) was placed around the cervix via vaginal examination by an trained health care provider. The required size was determined and confirmed through physical examination. The cervical cerclage was performed under general or spinal anaesthesia, and placed by experienced gynaecologists and using a Shirodkar or McDonald technique.^14^^,^^15^ Both interventions were initiated before 24 weeks GA, or before 16 weeks in case of a obstetric history indicated intervention, and stayed in situ until 36 weeks GA or until signs of imminent delivery such as (P)PROM or contractions. After initiation of the intervention, participants with a pessary or cerclage were treated and monitored similarly. Follow-up vaginal ultrasound, CL measurement or digital examination were not performed as part of standard procedure. In case of complaints, vaginal examination was advised to reposition or replace the pessary, if necessary. All participants were handled according to local and national protocols. This means vaginal swabs and Nugent scores were not performed routinely. Assessment of genital infections was only carried out in case of complaints or in selected cases with a high risk profile. Most participants were offered simultaneous treatment with progesterone (administered vaginally or parenterally) between 16 and 36 weeks of gestation in accordance with national guidelines on prevention of recurrent PTB. In case of imminent PTB, tocolysis and corticosteroids were offered to all.

### Outcomes

Outcome measures are in line with the core outcome set for studies on prevention of PTB.^16^ The primary outcome was PTB <32 weeks. Secondary outcomes included time from intervention to delivery; GA at delivery; PTB rates <24, 28, 34, and 37 weeks of gestation (overall, spontaneous and iatrogenic); PROM <36 weeks; use of tocolysis and/or corticosteroids during pregnancy; mode of delivery; maternal infections; maternal side effects. Perinatal outcomes were assessed through a composite of adverse perinatal outcomes (for details on definitions see study protocol).^12^ Outcomes were ascertained by qualified neonatologists as part of the clinical process and were not blinded.

Serious adverse events were defined as maternal death, life threatening events, events requiring hospitalization (for complications that were not inherent to pregnancy), events resulting in persistent or significant disability or incapacity, or any other serious or unexpected adverse event. Safety was monitored by the Data Safety and Monitoring Board (DSMB) through SAE monitoring and safety interim analyses. The first interim analysis was planned after all outcomes of 110 participants were available and the timing of subsequent interim analyses was determined by the DSMB. Monitoring of study procedures and protocol adherence was caried out by the trial bureau of the NVOG consortium.

### Patient and public involvement

The study was set up in collaboration with Care4Neo, a Dutch organisation for parents of premature infants, who provided full endorsement of the research proposal upon submisfunding body. For evaluation of interventions aimed at preventing PTB, we usion to ZonMw, the sed the core-outcome set,^16^ which was developed collaboratively with patient representatives.

### Statistics

We assumed an event rate of 20% for the primary outcome for cerclage based on literature available before initiation of this trial and used a non-inferiority margin of 10%. Using a one-sided alpha of 0.05 and power of 0.80, sample size was set at 400 participants (200 per arm).

Analysis was conducted according to a pre-specified statistical analysis plan (Supplement), adhering to the intention to treat principle. The primary outcome PTB <32 weeks was reported in incidence rates with relative risks, 95% confidence intervals (CI), and p-value using a binary general linear regression model (GLM) for both crude rates and adjusted rates (with centre and indication as fixed covariate). For the primary outcome, also the absolute risk difference was presented with 95% CI and two-sided p-value. The non-inferiority margin was set at 10%. This means that pessary treatment can be considered non-inferior to cerclage treatment of the upper limit is the 95% confidence interval of the absolute risk difference does not exceed 10%. If the absolute risk difference is lower than 10%, but the confidence interval exceeds 10%, non-inferiority would still not be proven. The Farrington-Manning test was used to test for non-inferiority. As a secondary analysis, a per protocol analysis was performed including participants whose allocated treatment was continued up to 36 weeks of gestation or until preterm delivery. In case of removal of intervention before 36 weeks of gestation or delivery for other reasons than PROM or delivery, participants were included in the per protocol analysis if their intervention was in situ for at least 80% of possible days from randomisation to 36 weeks or delivery (see statistical analysis plan for detailed description of the per protocol population). Secondary dichotomous outcomes were reported with incidence rates, relative risks, and 95% CLs. For continuous outcomes, means, standard deviations, and difference in means were reported together with the corresponding 95% CI and two-sided p-value. For highly skewed continuous outcomes, medians and interquartile ranges were reported and medians were compared using the non-parametric test for independent groups. Time from randomisation to delivery was evaluated by Kaplan–Meier estimates, using the log rank test.

We performed a pre-specified subgroup analysis on indication (obstetric history vs ultrasound guided indication) and on the number of previous preterm births (1 previous sPTB vs >1 previous PTB) with a test for interaction. Within the ultrasound guided indication subgroup, we also performed a predefined subgroup analysis on CL ≤ 15 mm and 15–25 mm and added a post-hoc analysis on the presence of funneling at randomisation.

### Ethics

This study was approved by the Medical Ethics Committee of the Amsterdam UMC (NL47362.018.13_AMC) while the boards of all participating centres approved local execution. This trial was registered at the International Clinical Trial Registry Platform (NL-OMON26958) and is reported according to the Consolidated Standards of Reporting Trials (CONSORT) checklist (Supplement).^17^ An independent Data Safety Monitoring Board (DSMB) provided oversight. Assessment for (serious) adverse events (SAE) was carried out directly after randomisation up until 30 days after delivery. Written informed consent was obtained from all participants included in the analyses.

### Role of funding source

The trial was funded by ZonMw (#837002406), the Netherlands Organisation for Health Research and Development. The funder had no influence on the design or conduct of the trial and was not involved in data collection, analysis, or writing of the manuscript.

## Results

Between March 26th 2014 and December 5th 2022, 261 participants were randomised to a cervical pessary (n = 133) or cervical cerclage (n = 128). After the third interim analysis which included complete data of 228 participants, the data and safety monitoring board advised the research group to suspend new inclusions due to safety concerns of perinatal morbidity and mortality in combination with the apparent challenge of establishing non-inferiority of pessary treatment.

**Fig. 1:**
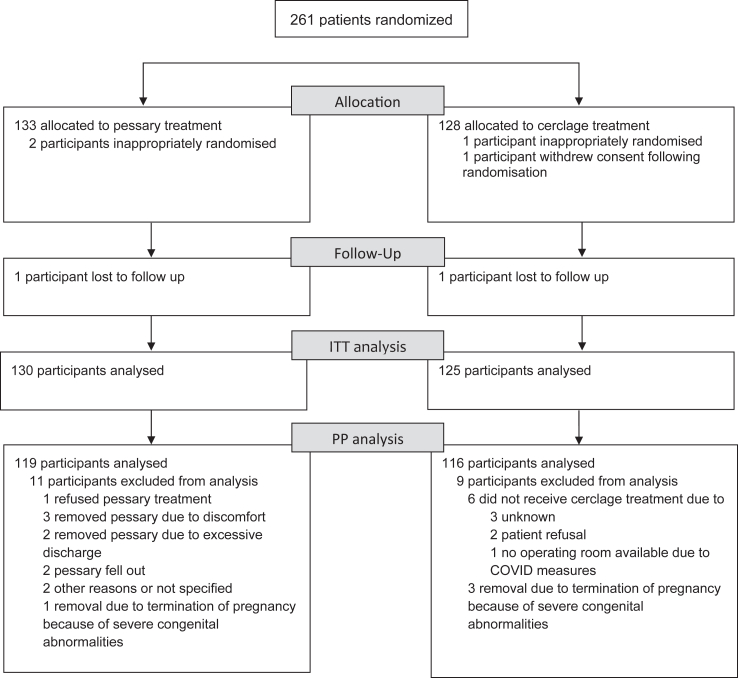
Flowchart of inclusion according to CONSORT guidelines (ITT: Intention-to-treat, PP: Per protocol).

In total, three participants were inappropriately randomised for not meeting the in- and exclusion criteria (pessary n = 2, cerclage n = 1) while one participant withdrew consent for data collection following randomisation (cerclage n = 1). The baseline characteristics of the remaining 257 participants are shown in Table 1. Two participants were lost to follow-up, so ultimately 255 participants were included in the intention to treat analysis (Fig. 1).

**Table 1:** Baseline characteristics.

	Pessary (n = 131)	Cerclage (n = 126)
**Maternal characteristics**		
Maternal age, years (mean, SD)	32.2 (4.8)	32.2 (5.4)
Body-mass index, kg/m^2^ (mean, SD)	26.2 (5.4)	26.8 (5.5)
Education		
Low^a^	10 (7.6%)	16 (12.7%)
Middle and high^b^	69 (52.7%)	49 (39.0%)
Unknown	52 (39.7%)	61 (48.8%)
Ethnicity		
White	65 (49.6%)	57 (45.2%)
Black (African, Afro-Caribbean, Afro-Surinam-Hindu)	40 (30.6%)	29 (23.0%)
Middle Eastern (North African, Turkish, Moroccan)	10 (7.6%)	19 (15.1%)
Asian (Asian, Indian, Pakistani)	5 (3.8%)	2 (1.6%)
Other	4 (3.1%)	2 (1.6%)
Unknown	4 (3.1%)	11 (8.7%)
History of cervical surgery (Conization/LLETZ^c^)	9 (6.8%)	7 (5.6%)
History of uterine surgery	7 (5.3%)	12 (9.5%)
Smoking (yes or quitted in first trimester)	15 (11.4%)	13 (10.3%)
Recurrent urinary tract infections (>3 per year)	2 (1.5%)	3 (2.4%)
Uterus anomaly	6 (4.6%)	5 (4.0%)
**Obstetric characteristics**		
Indication for intervention		
Obstetric history based indication <16 weeks	26 (19.8%)	30 (23.8%)
Ultrasound guided indication with cervix ≤ 25 mm	105 (80.2%)	96 (76.2%)
>1 previous preterm birth <34^+0^	22 (16.8%)	22 (17.5%)
History of curettage	38 (43.2%)	29 (39.2%)
Use of progesterone (vaginal or parenteral)	103 (78.6%)	104 (82.5%)
Pregnancy after IVF/ICSI^d^	8 (6.1%)	12 (9.5%)
Nugent score in first trimester performed	58 (44.6%)	51 (40.5%)
<4	32/58 (55.2%)	31/51 (60.8%)
4–7	14/58 (24.1%)	5/51 (9.8%)
>7	12/58 (20.7%)	15/51 (29.4%)
Vaginal swab performed	87 (66.4%)	80 (63.5%)
Negative	58/87 (66.7%)	48/80 (60.0%)
GBS	11/87 (12.6%)	12/80 (15.0%)
E coli	2/87 (2.3%)	0 (NA)
Trichomonas	0 (NA)	0 (NA)
Candida	5/87 (5.7%)	4/80 (5.0%)
Other	12/87 (13.8%)	16/80 (20%)
Gestational age (w + d) at randomization (median, IQR)	19^+4^ (16^+2^–21^+5^)	18^+4^ (15^+0^–21^+1^)
Cervical length at randomization (mm) (mean, SD)	20.1 (7.1)	19.8 (7.7)
Cervical length range		
0–15 mm	24 (21.2%)	26 (24.3%)
16–25 mm	81 (71.7%)	73 (68.2%)
>25 mm (primary indication only)	8 (7.1%)	8 (7.5%)
Funneling	50 (44.2%)	41 (38.0%)

a Primary school, prevocational secondary education (VMBO in Dutch).

b Senior general secondary education (HAVO in Dutch), pre-university secondary education (VWO in Dutch), secondary vocational education (MBO in Dutch), higher professional education (HBO in Dutch), and university education (WO in Dutch).

c LLETZ, large loop excision of the transformation zone.

d ISCI: intracytoplasmic sperm injections; IVF: in vitro fertilization.

Due to zero to few events in several of the participating centres, the adjustment for centre in the primary analysis was not possible. After combining comparable centres in the analysis (academic vs non-academic), no differences were seen compared to the crude results. Therefore, analysis was only adjusted for intervention indication.

The primary outcome of PTB <32 weeks occurred in 44/130 participants in the pessary group (33.8%) vs 30/125 in the cerclage group (24.0%) (aRR 1.4 95% CI 0.95–2.1, p = 0.09 and aRD 9.8% 95% CI −1.2 to 20.9). Given the upper limit of the 95% confidence interval exceeding the predefined 10% non-inferiority margin, non-inferiority of pessary treatment compared to cerclage treatment could not be proven. This was confirmed with the Farrington-Manning test (p = 0.51).

**Fig. 2:**
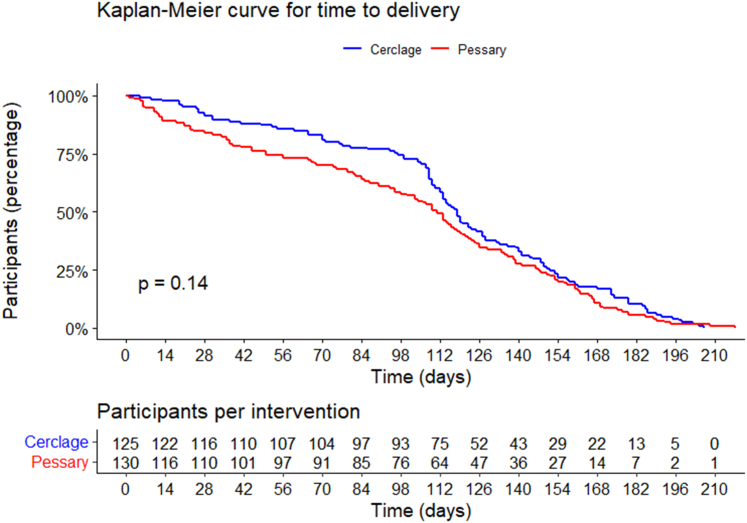
Kaplan Meier–Time from randomisation to delivery.

Primary and secondary outcomes are shown in Table 2. Rates of (s)PTB <28, sPTB <32, (s)PTB <34, and sPTB <37 weeks were significantly higher after pessary compared to cerclage. Median time from randomisation to delivery was 111.0 days after pessary (IQR 50.0–147.3) vs 118 days after cerclage (IQR 96.5–152.0) p = 0.2). The median GA at delivery was 36^+6^ (IQR 25^+4^–38^+6^) following pessary treatment vs 37^+5^ (IQR 33^+5^–39^+0^) following cervical cerclage (p = 0.2). The composite of adverse perinatal outcomes occurred in 42 cases after pessary compared to 29 cases in cerclage (32.2% vs 23.2%; RR 1.4 95% CI 0.93–2.1 p = 0.1) and consisted mainly of perinatal death (22.3% vs 14.4% RR 1.5 95% CI 0.9–2.6 p = 0.1). PROM <36 weeks occurred significantly more often in the pessary group (n = 54 42.2% vs n = 28 22.4%, RR 1.9 95% CI 1.2–1.7, p < 0.001). Maternal infections, vaginal blood loss and discomfort were comparable between groups. Vaginal discharge was reported more often after pessary than after cerclage (p < 0.001). The Kaplan–Meier curve for time from randomisation to delivery is shown in Fig. 2 (log rank p = 0.14).

**Table 2:** Primary and secondary outcomes.

	Pessary N = 130	Cerclage N = 125	RR (95% CI)	p-value	Absolute RD (95% CI)
**Primary outcome**					
PTB < 32 weeks (ITT) adjusted	44 (33.8%)	30 (24.0%)	1.4 (0.95–2.1)	0.09	
PTB < 32 weeks (ITT)^b^CRUDE	44 (33.8%)	30 (24.0%)	1.4 (0.95–2.1)	0.09	9.8 (−1.2 to 20.9)
PTB < 32 weeks (PP) adjusted	40/119 (33.6%)	27/116 (23.3%)	1.4 (0.94–2.1)	0.09	
PTB < 32 weeks (PP)^b^CRUDE	40/119 (33.6%)	27/116 (23.3%)	1.4 (0.95–2.2)	0.08	10.3 (−1.1 to 21.8)
**Obstetric outcomes**					
PTB < 37 weeks	65 (50.0%)	49 (39.2%)	1.3 (0.97–1.7)	0.08	
sPTB < 37 weeks	58 (44.6%)	37 (29.6%)	1.5 (1.1–2.1)	0.01	
PTB < 34 weeks	52 (40.0%)	32 (25.6%)	1.6 (1.1–2.3)	0.01	
sPTB < 34 weeks	48 (36.9%)	23 (18.4%)	2.0 (1.4–3.1)	<0.001	
PTB < 32 weeks	44 (33.8%)	30 (24.0%)	1.4 (0.95–2.1)	0.09	
sPTB < 32 weeks	41 (31.5%)	21 (16.8%)	1.9 (1.2–3.0)	0.006	
PTB < 28 weeks	41 (31.5%)	24 (19.2%)	1.6 (1.1–2.6)	0.02	
sPTB < 28 weeks	38 (29.2%)	18 (14.4%)	2.0 (1.2–3.4)	0.004	
PTB < 24 weeks	21 (16.2%)	14 (11.2%)	1.4 (0.77–2.7)	0.3	
sPTB < 24 weeks	20 (15.4%)	10 (8.0%)	1.9 (0.94–3.9)	0.07	
Time to delivery (days), median (IQR)	111.0 (50.0–147.3)	118.0 (96.5–152.0)		0.2	
GA at delivery (weeks), median (IQR)	36^+6^ (25^+4^–38^+6^)	37^+5^ (33^+5^–39^+0^)		0.2	
PROM^a^	54/128 (42.2%)	28/124 (22.6%)	1.9 (1.3–2.7)	<0.001	
Mode of delivery					
Vaginally	113 (86.9%)	102 (81.6%)	1.1 (0.96–1.2)	0.2	
Caesarean section	17 (13.1%)	23 (18.4%)	0.71 (0.40–1.3)	0.2	
Use of					
Tocolytic treatment	27/129 (20.9%)	16/123 (13.0%)	1.6 (0.91–2.8)	0.1	
Corticosteroids	35/128 (27.3%)	18/123 (14.6%)	1.8 (1.1–3.1)	0.01	
**Neonatal outcomes**					
Composite adverse neonatal outcome (ITT)	42 (32.3%)	29 (23.2%)	1.4 (0.93–2.1)	0.1	
Chronic Lung disease^b^	10 (7.8%)	7 (5.6%)	1.4 (0.54–3.5)	0.5	
IVH grade III or IV^b^	1 (0.8%)	1 (0.8%)	0.96 (0.06–15.2)	1.0	
PVL > grade 1^b^	2 (1.5%)	- (NA)	0.99 (0.96–1.0)	0.5	
NEC > stage 1^b^	2 (1.5%)	2 (1.6%)	0.96 (0.14–6.7)	1.0	
ROP^b^	4 (3.1%)	4 (3.2%)	0.97 (0.25–3.8)	1.0	
Patent ductus arteriosus	5 (3.9%)	3 (2.4%)	1.6 (0.39–6.6)	0.7	
Treated seizures	3 (2.3%)	4 (3.2%)	0.7 (0.17–3.2)	0.7	
Culture proven sepsis					
<72 h after birth (early)	- (NA)	2 (1.5%)	1.0 (0.99–1.0)	0.2	
>72 h after birth (late)	7 (5.4%)	6 (4.8%)	1.1 (0.4–3.2)	0.8	
Meningitis	2 (1.5%)	1 (0.8%)	1.9 (0.18–20.9)	1.0	
Perinatal death	29 (22.3%)	18 (14.4%)	1.5 (0.9–2.6)	0.1	
No live birth	23 (17.7%)	14 (11.2%)	1.6 (0.85–2.9)	0.1	
Stillbirth < 24 weeks	22 (16.9%)	14 (11.2%)	1.5 (0.81–2.8)	0.2	
Stillbirth > 24 weeks	1 (0.8%)	- (NA)	0.99 (0.97–1.0)	1.0	
Neonatal death > 24 weeks	6 (4.6%)	4 (3.2%)	1.4 (0.42–5.0)	0.7	
NICU admission	26 (20.0%)	16 (12.8%)	1.3 (0.89–2.0)	0.1	
Length of NICU admission (days), median (IQR)	13.0 (5.8–48.3)	26.0 (7.0–58.5)		0.4	
Lost to follow up	1 (0.8%)	1 (0.8%)	0.96 (0.06–15.2)	1.0	
**Maternal outcomes**					
Maternal mortality	0 (NA)	0 (NA)	NA	NA	
Maternal morbidity					
Infections					
Treated genital tract infections	7/126 (5.6%)	9/122 (7.4%)	0.75 (0.29–2.0)	0.6	
Treated urinary tract infections	20/128 (15.6%)	18/122 (14.8%)	1.1 (0.59–1.9)	0.8	
Chorioamnionitis	9/128 (7.0%)	7/123 (5.7%)	1.2 (0.48–3.2)	0.7	
Maternal side effects					
Vaginal discharge	34/124 (27.4%)	10/119 (8.4%)	3.3 (1.7–6.3)	<0.001	
Vaginal blood loss	19/127 (15.0%)	18/123 (14.6%)	1.0 (0.56–1.9)	0.9	
Discomfort or pain	20/126 (15.9%)	17/123 (13.8%)	1.1 (0.63–2.1)	0.6	

PTB: preterm birth, sPTB: spontaneous preterm birth.

NICU: neonatal intensive care unit, IQR: interquartile range, SD: standard deviation.

Primary outcome was assessed with binary general linear regression model (GLM) for both crude rates and adjusted rates (with centre and indication as fixed covariate) and the absolute risk difference is reported.

Secondary dichotomous outcomes reported in incidence rates, relative risks, and 95% CLs.

Skewed continuous outcomes reported in medians and interquartile ranges and were compared using the non-parametric test for independent groups.

a PROM: premature rupture of membranes <36 weeks.

b Severe respiratory distress syndrome (RDS) or Bronchopulmonary Dysplasia (BPD), NEC: Necrotizing Enterocolitis, PVL: Periventricular leukomalacia, ROP: Retinopathy of prematurity, IVH: Intraventricular Haemorrhage.

The per protocol analysis included 235 participants and showed comparable results. PTB <32 weeks occurred in 40/119 cases after pessary vs 27/116 cases after cerclage (33.6% vs 23.3% aRR 1.4, 95% CI 0.94–2.1, p = 0.09). Reasons for not following the study protocol are listed in Supplementary Table S2. In the pessary group, six participants received a cerclage following either failed placement (n = 1), pessary fell out (n = 2), excessive discharge (n = 1), or removal because labour was thought to start but did not continue after which a cerclage was placed (n = 2). In the cerclage group, three patients received a pessary because of patient refusal (n = 2) or unavailability of operating rooms due to COVID-19 measures (n = 1).

Table 3 shows the sub group analyses and the interaction between subgroups. A total of 56 participants had an intervention for cerclage indication based on obstetric history, while 199 were randomized following cervical length measurement. In the subgroup with an obstetric history based indication, PTB <32 weeks occurred in 11.5% of participants after pessary vs 20.0% after cerclage (RR 0.58 95% CI 0.16–2.0). In the ultrasound guided indication group, PTB <32 weeks was significantly higher after pessary compared to cerclage (39.4% vs 25.3% RR 1.6 95% CI 1.0–2.4). No effect modification was seen between a history indicated vs an ultrasound guided indication (P-value interaction 0.15). Exploratory analysis on PTB rates per indication are stated in Supplementary Table S1.

**Table 3:** Subgroup analyses on the primary outcome preterm birth (PTB) < 32 weeks.

	Pessary N = 130	Cerclage N = 125	Relative risk (95% CI)	p-value interaction term
**PTB <32 weeks (primary outcome)**				
Obstetric history based indication	3/26 (11.5%)	6/30 (20.0%)	0.58 (0.16–2.1)	0.15
Ultrasound guided indication	41/104 (39.4%)	24/95 (25.3%)	1.6 (1.0–2.4)	
1 previous PTB	33/104 (31.7%)	23/103 (22.3%)	1.6 (0.98–2.5)	0.91
>1 previous PTB	9/21 (42.9%)	7/22 (31.8%)	1.5 (0.56–4.0)	
**Within ultrasound guided indication group**				
Cervical length ≤15 mm	17/24 (70.8%)	8/25 (32.0%)	2.2 (1.2–4.1)	0.22
Cervical length 15–25 mm	24/79 (30.4%)	16/69 (23.2%)	1.3 (0.76–2.3)	
Funneling yes	26/49 (55.3%)	5/38 (13.2%)	4.2 (1.8–9.9)	0.002
Funneling no	13/46 (28.3%)	18/52 (34.6%)	0.8 (0.5–1.5)	

Supplementary Figure S1 displays the Kaplan–Meier curve for time to delivery among the obstetric history-based intervention subgroup (log-rank p = 0.58), while Supplementary Figure S2 shows the Kaplan–Meier curve for the ultrasound-guided indication subgroup (log-rank p = 0.35).

In the predefined subgroup analysis on number of previous preterm births, no differences between pessary and cerclage were seen within and between both subgroups (interaction p-value 0.91).

Within the subgroup of participants with ultrasound guided indication, we found a significant disadvantage of pessary over cerclage in participants with extreme cervical shortening ≤15 (70.8% vs 32.0% RR 2.2 95% CI 1.2–4.1). This disadvantage was not observed in participants with a CL of 15–25 mm (30.4% vs 23.2%, RR 1.3 95% CI 0.76–2.3). No effect modification was seen between groups (P-value interaction 0.22).

In the additional post-hoc analysis on the presence of cervical funneling at randomization, PTB <32 weeks was significantly higher after pessary compared to cerclage in participants with cervical funneling (55.3% vs 13.2% RR 4.2 95% CI 1.8–9.9). In participants without cervical funneling, this difference was not seen (28.3% vs 34.6%, RR 0.8 95% 0.5–1.5). Effect modification was seen between these subgroups (p = 0.002).

The incidence of serious adverse events was comparable between both groups and are displayed in Supplementary Table S3. A single participant in the pessary group developed pyelonephritis, necessitating hospitalization for intravenous antibiotic treatment. In one participant, cerclage removal was deferred until her primary caesarean section at 39 weeks of gestation, revealing a minor cervical laceration.

## Discussion

This study shows that pessary treatment did not meet the non-inferiority criteria when compared to cervical cerclage in individuals with a singleton pregnancy and a history of previous sPTB <34 weeks of GA with an indication for a cerclage.

This trial was halted prematurely due to safety concerns and futility reasons. The incidence of serious adverse events was comparable. However, PTB <34 weeks, NICU admission and perinatal death were all classified as adverse events and were reported more often in the pessary group, with higher rates of (extreme) PTB in this group. Even though the (safety) protocol stated that no interim analyses on intervention efficacy would be performed, the consequences of the mutual differences in neonatal and obstetric outcomes were considered to be of such relevance that it was decided to discontinue the trial prematurely from a safety perspective and because sufficient data appeared to be available to exclude the possibility of non-inferiority of pessary treatment. Previous studies has assessed the effectiveness of cervical pessary and cervical cerclage in various populations, including those with and without a history of sPTB or cervical shortening. Cerclage treatment is proven effective in reducing recurrent sPTB and composite perinatal morbidity and mortality within specific subgroups.^6^^,^^7^ Prior to this trial, a randomised controlled trial found no differences in pregnancy outcomes between cervical cerclage and pessary in individuals with a suspicion of cervical insufficiency.^9^ Another trial found promising results when comparing a cervical pessary to expectant management in a high risk population with cervical shortening.^8^ Nevertheless, subsequent systematic reviews concluded that the current available evidence does not support the use of cervical pessary as a preventive measure to improve obstetric and neonatal outcomes in singletons with cervical shortening, as no statistically significant benefit was observed.^7^^,^^10^^,^^11^ All stated that additional randomised trials were needed to determine optimal management strategies. Our results affirm that a cerclage is the preferred treatment in individuals with a singleton pregnancy and previous sPTB, particularly in cases of asymptomatic cervical shortening.

This study is one of the first RCT's to include a large sample size with a direct comparison evaluating the effectiveness of pessary vs cerclage in the prevention of recurrent PTB. Previous trials mainly focused on the effectiveness of a pessary or cerclage vs expectant management or progesterone treatment.^7^ Even though the sample size was not reached due to the suspension of new inclusions following the interim analyses, the results do show that a pessary is not non-inferior to cerclage. Furthermore, a distinction was made between the obstetric history based indication and ultrasound guided indication, which provides insight in the preventive mechanisms of the interventions in both groups.

A limitation of this study included that blinding was not possible due to the nature of interventions, which might introduce bias in regards to differences in management between interventions. As an example, even though discomfort did not differ between the groups, three pessaries were removed because of discomfort whereas no cerclages were removed for that reason. This could indicate a possible difference in the threshold for removal. Furthermore, we note that around 80% of our study population was randomised with an ultrasound-guided indication due to cervical shortening making the results less generalizable to a population with an obstetric history based indication. Simultaneous treatment with progesterone was indicated for all participants and since the use of progesterone was comparable between groups, we do not expect this influenced the effect difference between both interventions.

Four pregnancies in the cerclage group were terminated prior to reaching 24 weeks due to severe congenital abnormalities that were not apparent at the time of randomization. In contrast, only one pregnancy in the pessary group was terminated for similar reasons. These participants were included in the intention to treat analysis for that these anomalies were not visible at time of randomisation. They were excluded from the per protocol analysis.

Notably, PROM <36 weeks occurred significantly more often following pessary treatment compared to cerclage. Even though infections were reported comparable between groups, potentially there might be an inflammation-mediated mechanism such as dysbiosis of local inflammation due to long term placement of a foreign subject underlying this difference. In our study, Nugent scores for dysbiosis were only performed at the end of the first trimester in around 42% of participants, as it was not part of the study protocol and due to lack of uniform national guidelines on this subject. Out of participants who had a Nugent score performed, 40% required treatment, compared to 25–30% in previously performed studies on the effectiveness of cervical pessaries.^8^^,^^18^, ^19^, ^20^ The role of bacterial vaginosis on pregnancy outcomes or in intervention efficacy is unclear, but considering the high proportion requiring treatment, might deserve additional attention.^21^

A pessary is designed to support the lower uterine segment and prevent the cervix from shortening. However, we hypothesize that the effectiveness of a pessary might be dependent on cervical length at placement. In participants with cervical shortening, PTB <32 weeks GA was significantly higher after pessary, which was not seen in participants with a history indicated intervention who were randomised before cervical shortening could occur. This hypothesis is strengthened by the subgroup analysis of participants with a cervical length <15 mm, where a large disadvantage of pessary over cerclage was seen. There was no significant interaction between groups, yet this might also be due to low number of inclusions in the obstetric history indicated group. There was however a significant interaction between the groups with and without funneling, suggesting that a pessary is less effective in a funneling cervix. A previous study also found that placement of a pessary around a funneling short cervix resulted in a significantly higher rates of PTB <34 weeks compared to placement around a short cervix without funneling.^22^ Therefore, we hypothesize that the lower uterine segment is less supported by a pessary in case of a short or funneling cervix compared to cerclage and is therefore less effective in preventing PTB.

In conclusion, we did not prove non-inferiority of cervical pessary treatment compared to cerclage treatment in preventing recurrent PTB <32 weeks in high risk singletons with previous sPTB and an indication for a cerclage. Our findings are consistent with the notion that cervical cerclage is currently the preferred treatment option.

## Contributors

EP was the principal investigator at Amsterdam University Medical Centre, wrote the first drafts of the protocol and report, and is guarantor for the report. EP, BWM, BMK, MDZ, BK, MAL, BBB, CED and ALG represent the PC study group and were involved in conception and design of the study. ALG, CED, BK, MAL, BBB and MDZ were involved in the logistics of the study. MHK, BMT, ESA, MAB, MS, SJG, BBJH, YMM, SW, WMB, MH and MAO are local investigators at the participating centres. Underlying data were verified by ALG and CED, additionally to being monitored on a structural base during monitoring visits. ALG, CED, BMK, MCW conducted the analysis with consultation of EP and BWM. ALG, CED, MCW, MAO, BMK, BMW and EP drafted the manuscript, which follow the CONSORT checklist for reporting randomised trials. All authors received, read and approved the final draft of the manuscript.

## Data sharing statement

Study protocol, statistical analysis plan and informed consent form are available following publication. De-identified individual participant data that underlie the results reported in this article will be made available for the purpose of an individual participant data meta/analysis, to researchers whose proposed use of the data has been approved by an independent review committee identified for this purpose.

## Declaration of interests

The authors declare that the research was conducted in the absence of any commercial or financial relationships that could be construed as a potential conflict of interest. BMT reports receiving consulting fees for participation to the advisory board of Effik, Pierre-Favre and Sanofy, received payment for legal expert testimonies in malpractice cases in obstetrics and received a device to measure cervical softening from Pregnolia. BWM received an investigator grant from NHMRC. MAO received research grants for studies on the prevention of preterm birth, reports expenses paid for organization of preterm birth symposium, is chairman of the Fetal-Maternal Medicine board of the Dutch society of Obstetrics & Gynecology and chairman of the scientific committee of the Fetal-Maternal-Medicine Board. MAO and EP are board members of Stichting Stoptevroegbevallen, a non-profit organization supporting preterm birth research and received payments to the institution. EP received research grants for studies on prevention and treatment of preterm birth and the fetal microbiome.

## Appendix A Supplementary data

Supplementary Tables and Figures

C1-Onderzoeksprotocol-versie-6.0-dd-15-04-2020_clean

PC Study group

SAP_clean

s12884-017-1393-6

Consort_check
