# Supplementary material for: Pessary or cerclage (PC study) to prevent recurrent preterm birth: a non-inferiority, randomised controlled trial
Source: eClinicalMedicine. 2024 Nov 25;78:102945. doi: 10.1016/j.eclinm.2024.102945 (PMC11626620; doi:10.1016/j.eclinm.2024.102945)
Supplement: Supplementary Tables and Figures [file mmc1.docx]

**Supplementary appendix**

**Table of content**

- **Supplementary table 1:** Exploratory analysis on (s)PTB rates <24, < 28, <32, <34 and <37 weeks per obstetric history based indication or ultrasound guided indication
- **Supplementary table 2:** Details on use of intervention
- **Supplementary table 3:** Serious Adverse Events
- **Supplementary figure 1:** Kaplan Meier - Time from randomisation to delivery (history indicated subgroup)
- **Supplementary figure 2:** Kaplan Meier - Time from randomisation to delivery (ultrasound guided indicated subgroup)

| Obstetric history based indication |  | Pessary  N= 26 | Cerclage  N=30 |
| --- | --- | --- | --- |
| Composite outcome |  | 4 (15⸱4%) | 8 (26⸱7%) |
| PTB < 37 weeks |  | 8 (30⸱8%) | 8 (26⸱7%) |
| sPTB < 37 weeks |  | 6 (23⸱1%) | 5 (16⸱7%) |
| PTB < 34 weeks |  | 6 (23⸱1%) | 6 (20⸱0%) |
| sPTB < 34 weeks |  | 5 (19⸱2%) | 4 (13⸱3%) |
| PTB < 32 weeks |  | 3 (11⸱5%) | 6 (20⸱0%) |
| sPTB < 32 weeks |  | 3 (11⸱5%) | 4 (13⸱3%) |
| PTB < 28 weeks |  | 3 (11⸱5%) | 6 (20⸱0%) |
| sPTB < 28 weeks |  | 3 (11⸱5%) | 4 (13⸱3%) |
| PTB < 24 weeks |  | 2 (7⸱7%) | 4 (13⸱3%) |
| sPTB < 24 weeks |  | 2 (7⸱7%) | 2 (6⸱7%) |
| Ultrasound guided indication |  | **Pessary**  **N= 104** | **Cerclage**  **N=95** |
| Composite outcome |  | 38 (36⸱5%) | 21 (22⸱1%) |
| PTB < 37 weeks |  | 57 (54⸱8%) | 41 (43⸱2%) |
| sPTB < 37 weeks |  | 52 (50⸱0%) | 32 (33⸱7%) |
| PTB < 34 weeks |  | 46 (44⸱2%) | 26 (27⸱4%) |
| sPTB < 34 weeks |  | 43 (41⸱3%) | 19 (20⸱0%) |
| PTB < 32 weeks |  | 41 (39⸱4%) | 24 (25⸱3%) |
| sPTB < 32 weeks |  | 38 (36⸱5%) | 17 (17⸱9%) |
| PTB < 28 weeks |  | 38 (36⸱5%) | 18 (18⸱9%) |
| sPTB < 28 weeks |  | 35 (33⸱7%) | 14 (14⸱7%) |
| PTB < 24 weeks |  | 19 (18⸱3%) | 10 (10⸱5%) |
| sPTB < 24 weeks |  | 18 (17⸱3%) | 8 (8⸱4%) |
| PTB: preterm birth, sPTB: spontaneous preterm birth | | | |
| Supplementary table 1– Exploratory analysis on (s)PTB rates <24, < 28, <32, <34 and <37 weeks per obstetric history based indication or ultrasound guided indication | | | |

|  | Pessary  (n = 131 ) | Cerclage  (n = 126) |
| --- | --- | --- |
| Allocated treatment initiated | 129 | 120 |
| Lost to follow up | 1 | 1 |
| Removal of cerclage or pessary |  |  |
| According to study protocol^ | 120 | 116 |
| Gestational age > 36 weeks | 53 | 68 |
| Contractions or labour | 37 | 30 |
| Other reasons, required for delivery | 6 | 6 |
| (P)PROM* | 22 | 15 |
| Removed for other reason, but with >80% adherence | 3 | 1 |
| Not according to study protocol | 10 | 3 |
| Discomfort | 3 | 0 |
| Excessive discharge | 2 | 0 |
| Patient preference | 0 | 0 |
| Pessary fell out | 2 | 0 |
| Vaginal blood loss | 0 | 0 |
| Other | 2 | 0 |
| Removal of intervention because of termination of pregnancy due to severe congenital abnormalities | 1 | 3 |
| Supplementary Table 2 - Details on use of intervention | | |
| *(P)PROM: (premature) prelabour rupture of membranes | | |

| Serious Adverse Events | Pessary  *(n=130)* | Cerclage  *(n=125)* | Relative Risk  (95% CI) | p-value  subgroup |
| --- | --- | --- | --- | --- |
| Maternal death | 0 (NA) | 0 (NA) | NA (NA) | NA (NA) |
| Life threatening (at the time of event) to the mother | 0 (NA) | 0 (NA) | NA (NA) | NA (NA) |
| Hospitalization or prolongation for other than expected reason^#^ | 0 (NA) | 0 (NA) | NA (NA) | NA (NA) |
| Persistent or significant disability or incapacity of the mother | 0 (NA) | 0 (NA) | NA (NA) | NA (NA) |
| Severe congenital anomaly or birth defect of the neonate | 0 (NA) | 0 (NA) | NA (NA) | NA (NA) |
| Any other important medical event^$^ | 1 (0⸱8%) | 1 (0⸱8%) | 0⸱96 (0⸱06 – 15⸱2) | 0⸱98 |
| ^#^Requires hospitalization or prolongation of existing inpatients’ hospitalization other than expected obstetric complications (such as threatened premature labour, admissions due to labour or scheduled)  ^$^Any other important medical event that may not result in death, be life threatening, or require hospitalization, may be considered a serious adverse experience when, based upon appropriate medical judgement, the event may jeopardize the subject or may require an intervention to prevent one of the outcomes listed above⸱ | | | | |
| Supplementary Table 3– Serious Adverse Events | | | | |


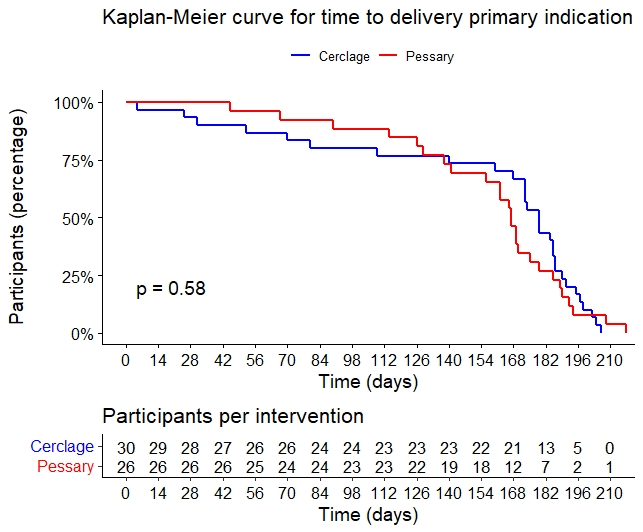


**Supplementary figure 1: Kaplan Meier - Time from randomisation to delivery (history indicated subgroup)**


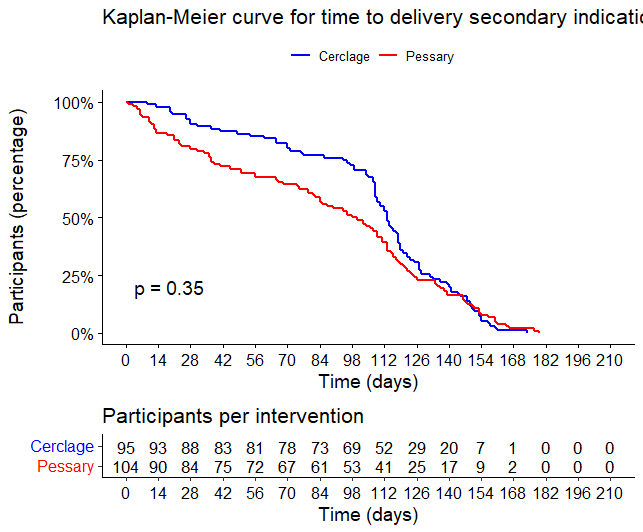


**Supplementary figure 2: Kaplan Meier - Time from randomisation to delivery (ultrasound guided indicated subgroup)**
