# Supplementary material for: Pessary or cerclage (PC study) to prevent recurrent preterm birth: a non-inferiority, randomised controlled trial
Source: eClinicalMedicine. 2024 Nov 25;78:102945. doi: 10.1016/j.eclinm.2024.102945 (PMC11626620; doi:10.1016/j.eclinm.2024.102945)
Supplement: C1-Onderzoeksprotocol-versie-6.0-dd-15-04-2020_clean [file mmc2.pdf]

Pessary or Cerclage to prevent preterm delivery in women with short cervical length and a history of preterm birth

- PC-study -

15 april 2020

Protocol version 6.0-

PROTOCOL TITLE: 'Pessary or Cerclage to prevent preterm delivery in women with short cervical length and a history of preterm birth'

**Protocol ID**	PC-Study ABR: NL: 47362.018.13
**Short title**	Pessary or Cerclage to prevent preterm delivery in women with short cervical length with a history of preterm birth
**Coordinating investigator/project leader/ principal investigator**<br><br>**Principle Investigator Switzerland**	Prof. dr. E. Pajkrt, gynaecologist<br>020 56661279, e.pajkrt@amsterdamumc.nl<br>Amsterdam UMC, location AMC<br>Postbus 22660<br>1100 DD Amsterdam Zuidoost<br><br>**Professor dr. Begoña Martinez de Tejada**<br>**Hopitaux Geneve, Switzerland**
**Sponsor (in Dutch: verrichter/opdrachtgever)**	Amsterdam UMC, location AMC
**Subsidising party:**	**ZonMW:** 80-83700-98-42055
**Independent expert:**	Dr. I.M. de Graaf, gynaecologist, Amsterdam UMC, location AMC

PROTOCOL SIGNATURE SHEET

Name	Signature	Date
**Head of the Department**		
**[Coordinating Investigator/Project leader/Principal Investigator]:**<br>**Prof. dr. E. Pajkrt, gynaecologist**		
**Investigator participating site**<br>**Name:**<br>**Site:**		

TABLE OF CONTENTS

1. STUDY DESIGN ... 17
2. STUDY POPULATION ... 18
2.1 Population (base) ... 15
2.2 Inclusion criteria ... 15
2.3 Exclusion criteria ... 15
2.4 Sample size calculation ... 16
3. TREATMENT OF SUBJECTS ... 20
3.1 Investigational product ... 20
4. INVESTIGATIONAL PRODUCT ... 21
4.1 Name and description of investigational product(s) ... 18
4.2 Summary of findings from non-clinical studies ... 18
4.3 Summary of findings from clinical studies ... 18
4.4 Summary of known and potential risks and benefits ... 19
5. NON-INVESTIGATIONAL PRODUCT ... 20
6. METHODS ... 21
6.1 Study parameters/endpoints ... 21
6.1.1 Main study parameter/endpoint ... 21
6.1.2 Secondary study parameters/endpoints (if applicable) ... 21
6.1.3 Long term follow-up ... 22
6.2 Randomisation, blinding and treatment allocation ... 22
6.3 Study procedures ... 22
6.4 Withdrawal of individual subjects ... 24
6.5 Premature termination of the study ... 24
7. SAFETY REPORTING ... 25
7.1 Temporary halt for reasons of subjectsafety ... 25
7.2 AEs, SAEs and SUSARs ... 25
7.2.1 Adverse events (AEs) ... 25
7.2.2 Serious adverse events (SAEs) ... 26
7.3 Follow-up of adverse events ... 27
7.4 Data Safety Monitoring Board (DSMB) ... 27
8. STATISTICAL ANALYSIS ... 31
8.1 Primary study parameter(s) ... 31
8.2 Secondary study parameter(s) ... 31
8.3 Other study parameters ... 31
8.4 Interim analysis (if applicable) ... 32
8.5 Regulation statement ... 33
8.6 Recruitment and consent ... 31
8.7 Compensation for injury ... 31
9. ADMINISTRATIVE ASPECTS, MONITORING AND PUBLICATION ... 33
9.1 Handling and storage of data and documents ... 33
9.2 Amendments ... 33
9.3 Annual progress report ... 33

9.4 End of study report 33
9.5 Public disclosure and publication policy 34
10. STRUCTURED RISK ANALYSIS 35
10.1 Potential issues of concern 35
10.2 Synthesis 36
11. REFERENCES 39

LIST OF ABBREVIATIONS AND RELEVANT DEFINITIONS

**AE**	**Adverse Event**
**AR**	**Adverse Reaction**
**CA**	**Competent Authority**
**CV**	**Curriculum Vitae**
**DSMB**	**Data Safety Monitoring Board**
**EU**	**European Union**
**GCP**	**Good Clinical Practice**
**IC**	**Informed Consent**
**IMP**	**Investigational Medicinal Product**
**IMPD**	**Investigational Medicinal Product Dossier**
**MREC**	**Medical research ethics committee; in Dutch: medisch ethische toetsing commissie (METC)**
**(S)AE**	**(Serious) Adverse Event**
**Sponsor**	**The sponsor is the party that commissions the organisation or performance of the research, for example a pharmaceutical company, academic hospital, scientific organisation or investigator. A party that provides funding for a study but does not commission it is not regarded as the sponsor, but referred to as a subsidising party.**
**GDPR**	**General Data protection Regulation**
**WMO**	**Medical Research Involving Human Subjects Act (in Dutch: Wet Medisch-wetenschappelijk Onderzoek met Mensen**

SUMMARY

Rationale: Preterm birth (PTB) is in quantity and in severity the most important pregnancy complication in obstetric care in the developed world. A cervical pessary and a cervical cerclage are both considered as potential preventive treatments for PTB in women with a history of preterm birth or women with a short cervical length.

Objective: To evaluate whether a cervical pessary can replace a cervical cerclage in women with at least one previous preterm birth and who are scheduled for cerclage, in terms of effectiveness and costs.

Study design: Non-inferiority, open-label multicenter randomized clinical trial with an economic analysis alongside it.

Study population: Asymptomatic women with a singleton pregnancy and a history of preterm birth before 34 weeks of gestation (GA) will be managed according to national guideline on previous PTB. (Richtlijn - Preventie recidief spontane vroeggeboorte, 2007, www.nvog.nl). According to local protocols, and in agreement with the national guideline mentioned above, all patients will be offered the use of progesterone from 16 weeks gestational age and cervical length measurements around 16, 18, 20 and 22 weeks of gestation. In women in whom cervical length is 25 mm or shorter (≤25mm) before 24 weeks of gestation, patient will be asked to participate in this randomized controlled trial. Women with a history of multiple premature deliveries and who are considered to be eligible for a primary cerclage before 16 weeks gestational age according to local protocols will also be asked to participate in this trial.

Intervention: Eligible women will be randomized before 24 weeks in case of previous preterm delivery and short cervical length to either a cervical pessary or cervical cerclage or before 16 weeks in case a primary cerclage is required. A pessary is made of soft and flexible silicone which can be easily placed or removed in an outpatient clinic and does not require anaesthesia. It is folded and put around the cervix by a simple vaginal examination without causing any pain. The cervical cerclage, also known as a cervical stitch, is used for the treatment of cervical incompetence. There are two main types of vaginal cerclage, McDonald or Shriodkar, both applied in a clinical setting in the operation theatre under general or spinal anesthesia. Both cervical pessary and cervical cerclage will be removed at 36 weeks of GA or until delivery.

Main study parameters/endpoints: The primary outcome will be delivery before 32 weeks (<32weeks). Secondary outcomes will be time from intervention to delivery, gestational age at delivery, preterm rate

birth before 24, 28, 34 and 37 weeks of gestation (overall and stratified by spontaneous or indicated delivery), premature rupture of membranes, use of tocolysis and/or corticosteroids during pregnancy, mode of delivery, maternal infections, maternal side effects and neonatal and maternal admissions. Perinatal outcome will be assessed through a composite of adverse perinatal outcome. . The composite outcome contains the following variables: Chronic lung disease, Intraventricular Haemorrhage (IVH) higher than grade II, Periventricular Leucomalacia (PVL) higher then grade I, Necrotizing Enterocolitis (NEC) higher then stage I, Retinopathy of Prematurity (ROP), Patent ductus arteriosus (PDA), treated seizures, early and late sepsis, neonatal meningitis, (intra-partum) stillbirth and death before discharge from the nursery. All components of the composite outcome will also be assessed individually.

Nature and extent of the burden and risks associated with participation, benefit and group relatedness:

BURDEN

Eligible women will be randomly allocated to receive either a cervical cerclage or pessary.

In case of a cervical cerclage it will be placed in situ before 24 weeks of gestation in women with previous preterm delivery and short cervical length or before 16 weeks of gestation in case a primary intervention is required, and will stay in situ up to 36 weeks gestation or until delivery, whatever comes first. The procedure involves occlusion of the cervix by means of a cervical suture or stitch, which is performed in the operation theatre under general or spinal anaesthesia and this is an invasive procedure. In case of a cervical pessary it will also be placed in situ before 24 weeks or before 16 weeks in case a primary intervention is required, and the pessary is a non-invasive method. The pessary is made of latex-free silicone which is soft and flexible. It is folded and put around the cervix by a simple vaginal examination without causing any pain. The pessary will remain within the vagina during pregnancy, until 36 weeks or until delivery, whatever comes first.

All women participating are enrolled after a transvaginal measurement for cervical length before 24 weeks of gestation or without cervical length measurement before 16 weeks of gestation in case of a primary intervention. *Arabin et al.* performed comparable research, showing in a questionnaire evaluation within the treatment group a general positive opinion of the treatment (Arabin, Halbesma, Vork, Hübener, & van Eyck, 2003). 75% of that treatment group would use the pessary again and would even recommend it to others. They reported that there might be some increase of vaginal discharge. If

there is an increase in discharge, women will be evaluated for vaginal infection and treated if necessary. Less common side effects during the use of a pessary are vaginal blood loss or pelvic pain. Cervical laceration as complication is rarely seen in the use of pessary, this chance seems smaller than 0.1%.. If recurrent or persistent blood loss, premature rupture of the membranes or contractions occur during the use of a pessary, the pessary should be removed.

Previous studies using the pessary have shown no foetal adverse effects.

BENEFIT

Cervical cerclage is applied in 1,000 women in the Netherlands annually. The cervical pessary is less invasive, and potentially has less complications, both from anaesthesia as from the cervical stitch itself. Potential complications of the stitch are infection premature rupture of membranes , cervical laceration or bleeding, all of which can result in immediate immature delivery with devastating effect for the child. The outcome of this study will indicate the relative effectiveness and the cost-effectiveness of cerclage and pessary.

INTRODUCTION AND RATIONALE

Preterm birth (PTB) is defined as delivery before 37 completed weeks of gestation (GA). In the Netherlands, PTB before 37 weeks occurs in 7.7% of the pregnancies, and in 1.3% of the pregnancies even before 32 weeks (Schaaf, Mol, Abu-Hanna, & Ravelli, 2011). Consequently, it affects 12.000 pregnancies per year. PTB can occur medically indicated, e.g. when the mother suffers severe hypertensive disease or when the foetus is severely growth restricted. About 75% of the cases however occur spontaneously, sometimes after a longer episode of preterm prelabour rupture of membranes (PPROM).

Preterm birth is a major contributor to perinatal mortality. Of all perinatal mortality 50-70% can be attributed to preterm birth. Similarly, spontaneous preterm birth is the leading cause of neonatal morbidity, mostly due to respiratory immaturity, intracranial haemorrhages and infections. These conditions can result in long term neurodevelopmental sequelae such as intellectual impairment, cerebral palsy, chronic lung disease, deafness and blindness. Thus, prevention of spontaneous preterm birth, with or without premature rupture of membranes, remains one of the biggest challenges in obstetric care.

An important risk factor for preterm birth is previous preterm birth. Women with a history of spontaneous preterm birth have a 20% risk of recurrence of spontaneous preterm birth before 37 weeks and 15% before 34 weeks (Esplin et al., 2008; Iams et al., 1998). In the Netherlands, pregnant women with a history of preterm birth before 34 completed weeks of gestation are currently managed according to a standard protocol that has been embedded in a national guideline (Otterlo, 2007).

This guideline includes the administration of intramuscular 17-hydroxy-progesterone or transvaginal progesterone from 16 gestational weeks' onwards. Moreover, cervical length is measured with transvaginal ultrasound at regular intervals between 16 and 24 weeks of GA. However, local guidelines may differ from the national guideline If a cervical length of 25 mm or less (≤25mm) is measured before 24 weeks GA, the recurrence risk of preterm birth is even more increased (Berghella et al. 2011), and this group of patients is offered a cervical cerclage. The risk of delivery before 32 weeks' gestation in the group with cerclage is 19,2% as compared to 29,5% in the group with no cerclage (RR 0,6 CI 0,48 – 0,91) (Berghella, Rafael, Szychowski, Rust, & Owen, 2011).

When a cerclage is indicated, eligible women are admitted to hospital to have the cerclage surgically inserted around the cervix before 24 weeks gestation. In women with a history of multiple preterm deliveries, a primary cerclage before 16 weeks of gestational age is necessary. This procedure is performed under general or spinal anaesthesia and takes approximately 30 minutes. Women stay overnight and are discharged the next day if no complications arise.

Annually approximately 100.000 multiparous women deliver in the Netherlands. Based on recent hospital numbers, we estimate that around 1% of these will have a cervical cerclage inserted, which adds up to 1.000 cerclages per year. We have recently reported that a cervical pessary is effective in the prevention of preterm birth in women with a twin pregnancy and a short cervical length (Liem et al., 2013), a finding that confirmed similar results among women with a singleton pregnancy and a short cervical length in Spain (Goya et al., 2012).

Since a cervical pessary can be positioned in an outpatient setting, and costs only 40 euros, it is potentially much more attractive than the cervical cerclage. In addition, a cervical pessary is a non-invasive method contrary to a cervical cerclage which is an invasive procedure. We hypothesize that the use of a cervical pessary will be equally effective in preventing preterm birth as cervical cerclage, and therefore propose to compare the effectiveness of the two approaches in a direct randomised comparison. The outcome of the proposed study will indicate the relative effectiveness of cervical

pessary for women with a singleton pregnancy and with previous preterm births and a short cervical length. Moreover, we will be able to compare the costs of both interventions. Since a pessary is much cheaper than the surgical application of a cerclage, implementation of this therapy will potentially yield a cost-reduction of 1.2 million euro per year.RELEVANCE FOR CLINICAL PRACTICE

As stated above, preterm birth is a severe health problem, in which prevention is the cornerstone of successful management. Cervical cerclage is applied in 1,000 women in the Netherlands annually. The cervical pessary is less invasive, and potentially has less complications, both from anaesthesia as from the cervical stitch itself. Potential complications of the stitch are infection, premature rupture of membranes, cervical laceration or bleeding, all of which can result in immediate immature delivery with devastating effect for the child. The outcome of this study will indicate the relative effectiveness and the cost-effectiveness of cerclage and pessary.

IMPLEMENTATION FEASIBILITY

Worldwide cooperation in this study will facilitate the implementation of results. Previously, we demonstrated that studies performed in our nationwide consortium were implemented even before theywere published or reached the guideline (van der Tuuk, Koopmans, Groen, Mol, & van Pampus, 2011). The results of the studies are incorporated in the national guidelines of KNOV (Dutch society for midwifes) and NVOG (Dutch society for obstetrics and gynaecology) soon after publication. Obstetrical guidelines are a national reference for local protocols, and midwives and gynaecologists are likely to follow the guidelines based on the results of the study. In addition, the results of the study will be published in international peer-reviewed journals.

If the results demonstrate non-inferiority of the pessary as compared to cerclage, due to statistical uncertainty we cannot rule out a small difference in favour of cerclage (equivalence limit). In addition, cerclage may prove to have a slightly lower success rate in preventing preterm birth, the question is whether women accept a slightly lower success rate of the pessary in order to avoid the burden and risks associated with the invasive cerclage procedure. To facilitate implementation, the study will therefore also include preference interviews with 20-30 women to address this question. The results will support decision making regarding adjustment of the guideline as well as clinicians in counselling these women in practice.

OBJECTIVES

Primary Objective:

The primary outcome will be delivery before 32 weeks.

Secondary Objectives:

Secondary outcome measures are time from intervention to delivery, gestational age at delivery, preterm birth rate before 24, 28, 34 and 37 weeks of gestation (overall and stratified by spontaneous or indicated), premature rupture of membranes, use of tocolysis and/or corticosteroids during pregnancy, mode of delivery, maternal infections (i.e. two measurement of maternal temperature above 37,8 degrees Celsius at a one hour interval and a maternal pulse > 100 beats per minute requiring treatment with antibiotics), maternal side effects (i.e. vaginal discharge, bleeding, discomfort, dyspareunia, cervical laceration) and both neonatal and maternal admissions. Perinatal outcome will be assessed through a composite of adverse perinatal outcome.. This composite outcome contains:

- Chronic lung disease
- Intraventricular Haemorrhage (IVH) grade III and IV: defined as hemorrhage in the germinal matrix, ventricles, or cerebral parenchyma; observed on ultrasound examination or MRI. Ultrasound examination is routinely performed in all neonates born prior to 32 weeks GA, or in neonates with neurologic symptoms.
- Periventricular Leucomalacia (PVL) higher then grade I: Periventricular lucency in the white matter
- Necrotizing Enterocolitis (NEC) higher then stage I: defined as the presence of the characteristic clinical features of abdominal distention, with or without rectal bleeding, and abdominal radiographic finding associated with pneumatosis intestinalis (this last finding is an abnormal gas pattern with dilated loops consistent with ileus)
- Retinopathy of Prematurity (ROP)
- Patent ductus arteriosus (PDA)
- Treated seizures
- Early and late sepsis: classified as suspected or proven (caused by any pathogen) and defined as a neonatal infection with cardiorespiratory instability or a positive blood culture caused by any pathogen. Clinical infection will include symptoms like positive findings on clinical exam, imaging,

or laboratory tests. Laboratory signs of infection will be increased CRP, leukocytosis or leukocytopenia.

- Neonatal meningitis: classified as suspected or proven (caused by any pathogen). Clinical infection will include symptoms like positive findings on clinical exam, imaging, or laboratory tests. Laboratory signs of infection will be increased CRP, leukocytosis or leukocytopenia.
- (Intrapartum) stillbirth
- Death before discharge from the nursery
- In addition, a cost-effectiveness analysis will be performed that will be reported separately from the primary report on the randomised trial.

STUDY DESIGN

Study design:

Non-inferiority, multicenter randomized controlled study. We will investigate women with a singleton pregnancy. For singleton pregnancies, the standard intervention is a cerclage, so pessary will be compared with a cerclage.

Setting:

Compare the costs and effectiveness of cervical pessary versus cervical cerclage in the prevention of preterm birth in asymptomatic women with a singleton pregnancy, a history of preterm birth and a short cervical length (25 mm or less) before 24 weeks' gestation and women with a history of multiple preterm deliveries who are considered for a primary cerclage before 16 weeks gestation.

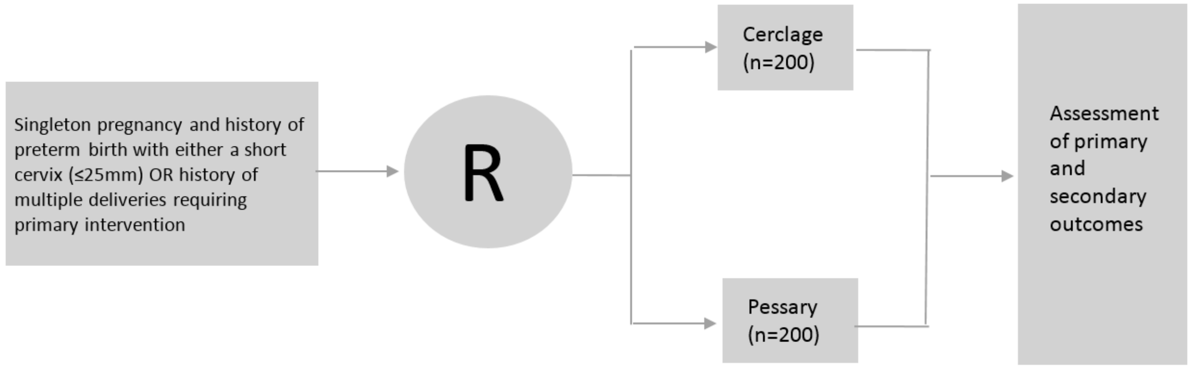

1. STUDY POPULATION

1.1 Population (base)

Asymptomatic women with a singleton pregnancy and a history of preterm birth before 34 weeks' gestation and women with a history of multiple preterm births will be managed according to the guideline on previous preterm birth (Otterlo, 2007). All patients will be treated according to local protocols. Most patients will be offered the use of progesterone and cervical length measurements around 16, 18, 20 and 22 weeks' gestation, however the exact treatment protocol could vary between different hospitals. In women in whom the cervix is 25 mm or shorter before 24 weeks' gestation, the patient will be asked to participate in a randomized controlled trial. Women who are considered for a primary intervention before 16 weeks gestation, will also be asked to participate in this trial.

1.2 Inclusion criteria

In order to be eligible to participate in this study, a subject must meet all of the following criteria:

1. Singleton pregnancy

2. History of preterm birth before 34 weeks of gestation

AND

3. Cervical length of 25mm or less on transvaginal ultrasound before 24 weeks of GA

OR

Indication for primary cerclage before 16 weeks in current pregnancy based on obstetric history, according to local protocol

4. Written informed consent

1.3 Exclusion criteria

A potential subject who meets any of the following criteria will be excluded from participation in this study:

1. Maternal age less than 18 years
2. Inability to give informed consent
3. Placenta praevia

4. Vasa praevia
5. Premature Prelabour Rupture of the Membranes (PPROM)
6. Cervical dilatation ≥3cm
7. Cervical length <2mm
8. Identified major congenital abnormalities: Major foetal abnormalities are defined as those that are lethal or require intensive prenatal care or postnatal surgery.
9. Women with clinical signs of chorioamnionitis or signs of intra uterine infection, defined as a temperature > 37,8 degrees Celsius, maternal tachycardia > 100 beats per minute and fetal tachycardia > 160 beats per minute, without any other focus of infection.

1.4 Sample size calculation

We plan to evaluate the non-inferiority of a cervical pessary as compared to cervical cerclage. We assume an event rate of 20% for the primary outcome, i.e. delivery before 32 weeks, for cerclage based on current literature. We will use a non-inferiority margin of 10%. This is equivalent to saying that pessarium is non-inferior to cerclage when the upper limit of the 95% confidence interval of the event rate of the primary outcome in the pessarium group is less than 30%. Using a one-sided alpha of 0.05 and power of 0.80 we need 2 groups of 200 women.

2. TREATMENT OF SUBJECTS

2.1 Investigational product

The pessary is made of silicone, which is soft and flexible. It is folded and put around the cervix by a simple vaginal examination without causing any pain. The pessary will remain within the vagina during pregnancy, until 36 weeks or until delivery, whatever comes first. Arabin et al. performed comparable research, showing in a questionnaire evaluation within the treatment group a general positive opinion of treatment (Arabin et al., 2003). 75% of women treated with a pessary would use it again and would even recommend it to others. They reported that there might be some increase of vaginal discharge. If there is an increase in discharge, women will be evaluated for vaginal infection and treated if necessary. Although the exact mechanism of action of the cervical pessary is not known, it has been hypothesized that the pessary encompasses the cervix and changes the inclination of the cervical canal. By relieving direct pressure on the internal cervical ostium it distributes the weight of the pregnant uterus onto the vaginal floor, retrosymphyseal osteomuscular structures and Pouch of Douglas (Vitsky, 1961). Hence, it may prevent premature dilatation of the cervix and premature rupture of the membranes. Another possible explanation is that due to the encompassed cervix the cervical canal is compressed and this might prevent deterioration or loss of the cervical mucus plug (CMP). During pregnancy the cervix normally stays tightly closed with a CMP sealing the opening. The role of the cervical mucus plug as an immunological gatekeeper, protecting the foetoplacental unit against infection from the vagina, may potentially play an important role in preventing ascending infections leading to preterm delivery. (Becher, Adams Waldorf, Hein, & Uldbjerg, 2009; Hein, Helmig, Schønheyder, Ganz, & Uldbjerg, 2001). Our group and the participating hospitals already are experienced users of the pessary thanks to the ProTwin trial which was just finished (Liem et al., 2013).

The most reported side effect of a pessary is vaginal discharge. Less common side effects during the use of a pessary are vaginal blood loss or pelvic pain. In addition, the complication of cervical laceration has been rarely seen, this chance seems to be smaller than 0.1%. If recurrent or persistent blood loss, premature rupture of the membranes or contractions occur during the use of a pessary, the pessary should be removed.

3. INVESTIGATIONAL PRODUCT

3.1 Name and description of investigational product(s)

Arabin cerclage pessary – produced by dr. Arabin & Co. GmbH
Vaginal pessaries have been used to prevent preterm birth since 1959 (Vitsky, 1961). Besides occlusion of the cervical os by compression, the pessary might prevent preterm birth by relieving the internal cervical os from direct pressure by distributing the weight of the pregnant uterus onto the vaginal floor and thus preventing premature dilatation of the cervix and premature rupture of the membranes. This effect may be amplified as the pessary changes the angle of the cervical canal. The most frequently applied pessary in the Netherlands is the Arabin pessary (Arabin et al., 2003). Different sizes are available. Per patient, the appropriate size has to be estimated by gynaecological exam. Hence, it may prevent premature dilatation of the cervix and premature rupture of the membranes.
Another possible explanation is that due to the encompassed cervix the cervical canal is compressed and this might prevent deterioration or loss of the cervical mucus plug (CMP). During pregnancy the cervix normally stays tightly closed with a CMP sealing the opening. The role of the cervical mucus plug as an immunological gatekeeper, protecting the foetoplacental unit against infection from the vagina, may potentially play an important role in preventing ascending infections leading to preterm delivery (Becher et al., 2009; Hein et al., 2001)

3.2 Summary of findings from non-clinical studies

Not applicable.

3.3 Summary of findings from clinical studies

The only RCT of pessary prophylaxis compared a pessary to cerclage. The type of pessary used, the entry criteria and the method of randomisation, were not well described (Förster et al. 1986). The trial recruited 112 patients in the cerclage group and 130 in the pessary group. Mean gestational age at initiation of the therapy was 27 weeks. Both methods were equal in their effects, but in view of the poor quality of the trial and the late gestational age at start of treatment, these results have little significance.
A prospective cohort study by Acharya et al. also used an double ring shaped pessary (Arabin-pessary) to treat cervical incompetence in women with a cervical length ≤ 25mm, before 30 weeks (Acharya et

al., 2006). The study showed that in 55 percent of the patients, the pregnancy could be postponed to 34 weeks or more. All other reports have been of uncontrolled case series or non-randomized comparative studies.

A double ring shaped pessary is at present the most popular pessary. It has been evaluated in one cohort study (Arabin et al., 2004). This study showed in women with a twin pregnancy a mean gestational age at delivery 35+6 (33-37+4) in the pessary group (n=23) and 33+2 (24+4-37+2) in control group (n-23) (p=0.02). From the 23 women with a pessary, no one delivered before 32 weeks versus 8 women in the control group (P < .001, RR 0.12, 95% CI 0.02 to .88). Based on this study, the Arabin pessary is widely used in The Netherlands and Germany with the manufacturers selling around 2000 per year.

In April 2012 the results of the PECEP-trial were published (Goya et al., 2012). This RCT included women with singleton pregnancies who have at ultrasound examination at 18+0 to 22+6 weeks of gestation a short cervical length. Patients were randomized to a pessary or no intervention. The proportion of spontaneous deliveries before 34 weeks, the trial’s primary endpoint, was reduced in women who received a cervical pessary (6% vs. 27%; odds ratio 0·18, 95% CI 0·08–0·37). These results are very impressive and promising.

Within the Dutch obstetric research consortium, we just finished the ProTwin trial studying the effectiveness of pessary in twin pregnancies (Liem et al., 2013). Although there was no treatment effect of pessary in the unselected group of women with a twin pregnancy, in the pre-specified subgroup of women with a cervix less than the 25th percentile (38 mm), the pessary group significantly reduced preterm delivery rates (11% vs. 25%, RR 0.44; 95%CI 0.20-0.98 for delivery less than 32 weeks). More important the poor neonatal outcome rate was substantially lower as compared to no intervention (7% vs. 30%, RR 0.23 95% CI; 0.09-0.60).

3.4 Summary of known and potential risks and benefits

The pessary might increase vaginal discharge (Arabin et al., 2003; Goya et al,. 2016). If there is an increase in discharge, women will be evaluated for vaginal infection and treated if necessary. Less common side effects during the use of a pessary are vaginal blood loss or pelvic pain. In addition, the complication of cervical laceration has been rarely seen, this chance seems to be smaller than 0.1%. If recurrent or persistent blood loss, premature rupture of the membranes or contractions occur during the

use of a pessary, the pessary should be removed. Previous studies using the pessary have shown no foetal adverse effects.

4. NON-INVESTIGATIONAL PRODUCT

Cervical cerclage:

During normal pregnancy, the cervix (neck of the womb) of the uterus is tightly closed with a cervical mucus plug sealing the opening. Towards the end of pregnancy the cervix starts to shorten and progressively becomes softer preparing for delivery. Sometimes, the cervix starts to shorten too early, causing dilatation and potentially preterm delivery. It has been hypothesized that impairment of the mucous plug, for example by contractions, rupture of membranes or effacement of the cervix, can lead to an ascending infection and preterm delivery. Consequently, mechanical occlusion of the cervix, may reduce the risk of preterm birth. Mechanical prevention of preterm birth by occlusion of the cervix can be accomplished by a cervical cerclage. This method is a well-known surgical procedure carried out during pregnancy proposed by Shirodkar in 1955 and by McDonald in 1957. Both procedures involve occlusion of the cervix by means of a cervical suture or stitch which are performed under general or spinal anaesthesia.

Side effects and complications of cerclage include increased vaginal discharge, cervical bleeding, infection, premature rupture of membranes and cervical laceration.

5. METHODS

5.1 Study parameters/endpoints

5.1.1 Main study parameter/endpoint

The primary outcome will be delivery before 32 weeks.

5.1.2 Secondary study parameters/endpoints (if applicable)

Secondary Objectives:

Secondary outcome measures are time from intervention to delivery, gestational age at delivery, preterm birth rate before 28, 32, 34 and 37 weeks of gestation (overall and stratified by spontaneous or indicated), premature rupture of membranes, use of tocolysis and/or corticosteroids during pregnancy, mode of delivery, maternal infections (i.e. two measurement of maternal temperature above 37,8 degrees Celsius at a one hour interval and a maternal pulse > 100 beats per minute requiring treatment with antibiotics), maternal side effects (i.e. vaginal discharge, bleeding, discomfort, dyspareunia, cervical laceration) and both neonatal and maternal admissions. Perinatal outcome will be assessed trough a composite of adverse perinatal outcome. This composite outcome contains:

- Chronic lung disease
- Intraventricular Haemorrhage (IVH) grade III and IV: defined as hemorrhage in the germinal matrix, ventricles, or cerebral parenchyma; observed on ultrasound examination or MRI. Ultrasound examination is routinely performed in all neonates born prior to 32 weeks' gestation, or in neonates with neurologic symptoms.
- Periventricular Leucomalacia (PVL) higher then grade I: Periventricular lucency in the white matter
- Necrotizing Enterocolitis (NEC) higher then stage I: defined as the presence of the characteristic clinical features of abdominal distention, with or without rectal bleeding, and abdominal radiographic finding associated with pneumatosis intestinalis (this last finding is an abnormal gas pattern with dilated loops consistent with ileus)
- Retinopathy of Prematurity (ROP)
- Patent ductus arteriosus (PDA)
- Treated seizures
- Early and late sepsis: classified as suspected or proven (caused by any pathogen) and defined as a neonatal infection with cardiorespiratory instability or a positive blood culture caused by any

pathogen. Clinical infection will include symptoms like positive findings on clinical exam, imaging, or laboratory tests. Laboratory signs of infection will be increased CRP, leukocytosis or leukocytopenia.

- Neonatal meningitis: classified als suspected or proven (caused by any pathogen). Clinical infection will include symptoms like positive findings on clinical exam, imaging, or laboratory tests. Laboratory signs of infection will be increased CRP, leukocytosis or leukocytopenia.
- (Intra-partum) stillbirth
- Death before discharge from the nursery

5.1.3 In addition, a cost-effectiveness analysis will be performed that will be reported separately from the primary report on the randomised trial Long term follow-up

If sufficient budget is awarded the possibilities to perform a long-term follow-up will be assessed. This will be added to the trial documents using an amendment. Permission to approach patients for follow-up research will be asked via the informed consent.

5.2 Randomisation, blinding and treatment allocation

GCP trained nurses will counsel patients, ask informed consent, perform randomization and collect data. Randomization will be centrally controlled using an on-line computerised randomisation service, once patient data have been entered in a web-based database. Centres will be able to access the randomisation service 24hr/day. Subjects will be randomized in a 1:1 ratio to cervical cerclage and pessary. Randomization will be stratified by indication for type of cerclage (primary or secondary) and centre (to prevent any imbalance between groups in aspects of maternal or neonatal care that may differ between centres). We will apply block randomisation with a variable block size Due to the type of interventions this study will not be blinded.

5.3 Study procedures

Eligible women will be randomly allocated to receive either a cervical cerclage or a cervical pessary. Both will be placed in situ before 24 weeks, or before 16 weeks in case of a primary intervention, and will stay in situ up to 36 weeks gestation or until delivery, whatever comes first.

Before entry into the study, the investigator or an authorized member of the investigational staff must explain to potential subjects the aims, methods, reasonably anticipated benefits, and potential hazards of the study. Subjects will be informed that their participation is voluntary and that they may withdraw consent to participate at any time. They will be informed that choosing not to participate will not affect the care the subject will receive. The research nurse who will obtain the informed consent can give more information.

Each subject must give written consent. The subject will be given sufficient time to read the patient information and the informed consent form and gets the opportunity to ask questions. An independent physician will be accessible for any question the subjects may have. The consent form must be signed before any study-related activity can take place. A copy of the informed consent form must be given to the subject.

In all women, we will measure cervical length prior to randomisation except in those who are considered for primary intervention before 16 weeks of gestation. Transvaginal ultrasonography will be carried out with a 5-MHz transducer. The probe will be placed in the anterior fornix of the vagina and a sagittal view of the cervix, with the echogenic endocervical mucosa along the length of the canal, will be obtained. The callipers will be used to measure the distance between the triangular area of echodensity at the external os and the V-shaped notch at the internal os. The presence or absence of funnelling at the internal os will also be recorded.

All women participating will be enrolled after transvaginal measurement for cervical length before 24 weeks or before 16 weeks when primary intervention is required. Next to this research intervention cases are treated according to the local protocol in the participating clinics and other interventions i.e. tocolysis and corticosteroids in case of a threatened preterm birth can be carried out as usual. Furthermore no extra interventions will be needed.

The pessary is made of silicone which is soft and flexible. It is folded and put around the cervix by a simple vaginal examination without causing any pain. It is important that the pessary is placed by a care giver with expertise to ensure careful placement of the pessary. In case of complaints, examination of the patient is advised to reposition the pessary or to replace the pessary with another size if necessary. The cervical cerclage will be placed under general or spinal anaesthesia.

5.4 Withdrawal of individual subjects

Subjects may withdraw at any time or be withdrawn by the investigator if the woman violates the study plan or for administrative and /or safety reasons. Patients who withdraw from the study will remain in their treatment group for the intent-to-treat analysis. Every effort will be made to obtain complete information on each patient randomized. The only reason for not obtaining complete information is that the patient was lost to follow up or that she withdraws consent to access her medical chart after delivery. Once a woman has been randomized, even though she stops taking the study medication/device for any reason, follow-up will be continued including the planned visits, maternal and fetal surveillance. If a woman refuses to complete her follow-up visits with the research nurse, the research nurse will confirm permission to consult her hospital chart in order to be able to complete information on the primary outcome of the study. In this case, her data will be considered in the final analysis.

5.5 Premature termination of the study

The study can be stopped prematurely based on recommendations of the Data Safety Monitoring Board (7.4 and 8.2). The DSMB can advise to stop the trial at any moment when the safety of the patients is considered to be in danger. These reasons should be noted. When the principal investigator deviates from the advice from the DSMB this will be reported to the MREC.

6. SAFETY REPORTING

6.1 Temporary halt for reasons of subjectsafety

In accordance to section 10, subsection 4, of the WMO, the sponsor will suspend the study if there is sufficient ground that continuation of the study will jeopardise subject health or safety. The sponsor will notify the accredited MREC without undue delay of a temporary halt including the reason for such an action. The study will be suspended pending a further positive decision by the accredited MREC. The investigator will take care that all subjects are kept informed.

6.2 AEs, SAEs and SUSARs

6.2.1 Adverse events (AEs)

Adverse events are defined as any undesirable experience occurring to a subject during the study, whether or not considered related to the investigational product / the experimental intervention. Due to the fact that the population is pregnant with a notable risk of immature and premature birth, specific SAE's are to be expected and will not be reported through Toetsing Online. They will be reported to the MREC by line listing every six months.

For this protocol only the following adverse events will be collected:

- Perinatal death
- Severe neonatal morbidity
- Preterm delivery before 34 weeks
- Necrosis of the cervix as a result of the pessary
- Hospitalisation or prolongation of existing inpatients' hospitalisation of the mother due to threatened preterm birth or other pregnancy induced pathology like pregnancy induced hypertension, pre-eclampsia, hydronephrosis etc.

Adverse events of special interest regardless relationship to investigational product occurring from the first study-related procedure until 30 days after delivery. Adverse events occurring after 30 days should also be reported if considered at least possibly related to the investigational medicinal product by the investigator

The AEs must be documented in an AE form and the outcome must be followed-up until the return to normal or consolidation of the patient's condition.

6.2.2 Serious adverse events (SAEs)

A serious adverse event is any untoward medical occurrence or effect that at any dose:

- results in maternal death;
- is life threatening (at the time of the event);
- requires hospitalisation or prolongation of existing inpatients' hospitalisation other than named above in the AE listing
- results in persistent or significant disability or incapacity of the mother;
- Any other important medical event that may not result in death, be life threatening, or require hospitalization, may be considered a serious adverse experience when, based upon appropriate medical judgement, the event may jeopardize the subject or may require an intervention to prevent one of the outcomes listed above.

The following events are not considered to be a Serious Adverse Event:

- Admissions due to premature labour or threatened premature labour. Premature delivery before 34 weeks has to be reported as AE.
- Admissons due to labour or scheduled delivery/ caesarean section > 34 weeks.
- Hospitalization for a procedure that was planned prior to study participation (i.e. prior to registration or randomization). This should be recorded in the source documents. Prolonged hospitalization for a complication of such procedures remains a reportable serious adverse event.
- Prolonged hospitalization for technical, practical, or social reasons, in absence of an adverse event.

Serious Adverse Events (SAEs) will be reported from the first study-related procedure until 30 days after delivery. Serious Adverse events occurring after 30 days should also be reported if considered at least possibly related to the investigational medicinal product by the investigator.

The expedited reporting will occur not later than 15 days after the sponsor has first knowledge of the serious adverse reactions. SAEs that result in death or are life threatening should be reported expedited. The expedited reporting will occur not later than 7 days after the responsible investigator has first knowledge of the adverse reaction. This is for a preliminary report with another 8 days for completion of the report.

A Serious Adverse Device Effect is an adverse effect of the device that has resulted in any of the consequences characteristic of a serious adverse event. The project group will evaluated if the reported SAE is a SADE.

6.3 Follow-up of adverse events

All (serious) adverse events will be followed clinically until they are resolved , or until a stable situation has been reached. Depending on the event, follow up may require additional tests or medical procedures as indicated, and/or referral to the general physician or a medical specialist.
Follow up information on SAE's should be reported monthly until recovery or until a stsable situation has been reached. The final outcome of the SAE should be reported on a final SAE report from provided.
Subjects withdrawn from the study due to one of the AEs will be followed at least until the outcome has determined even if it implies that the follow-up continues after the patient has left the trial.

6.4 Data Safety Monitoring Board (DSMB)

The members of the independent data safety monitoring board are:

(1) Dr J.J. Duvekot (chair): Hans Duvekot is gyaecologist-perinatologist at the dept. of obstetrics, Sophia Children's Hospital, Erasmus Medical Center Rotterdam. He has been involved in several multicentre trials within the NVOG Consortium.

(2) Professor K.C.B. Roes: Kit Roes is professor in Biostatistics at the Radboud University Medical Center, and a research interest in statistical design and analysis of clinical trials and trials in small populations. He served as member and chair on multiple DSMBs.

(3) Dr H.I.J. Wildschut: Hajo Wildschut was active in clinical care as a consultant gynaecologist-perinatologist until his retirement in 2016. He has special interest in the fields of quality of care, epidemiology, screening and informed consent.

(4) Professor H.W. Bruinse: Hein Bruinse was professor in Clinical Obstetrics at the UMC Utrecht until he retired in 2010. He has been project leader of five randomised clinical trials, of which one study within the NVOG Consortium.

(5) Dr J.H. van der Lee (secretary): Hanneke van der Lee is specialist in epidemiological research in general practice and paediatrics at the Academic Medical Center, Amsterdam. Her main interests are clinimetrics of health outcomes in RCTs, and RCT methodology related to optimal sample sizes and Data Monitoring Committees.

The advice(s) of the DSMB will be communicated to the principal investigator of the study, the trial methodologist and the NVOG Consortium's clinical trials unit. When an interim-analysis is executed a copy will also be sent to the independent methodologist. In case of material recommendations (e.g. when suspension or termination of the study is recommended), these will also be communicated to the sponsor's representative (e.g. head of department). Should the principle investigator decide not to fully implement the advice of the DSMB, the principle investigator will send the advice to the reviewing MREC, including a note to substantiate why (part of) the advice of the DSMB will not be followed.

7. STATISTICAL ANALYSIS

7.1 Primary study parameter(s)

Data will initially be analysed according the intention to treat method and outcome assessors will be blinded to treatment allocation. We will assess the differences between the two groups by calculating the relative risk of the the main outcome with a log-binomial mixed model. Stratification by centre will be accounted for with a random intercept for each centre. When appropriate, numbers needed to treat will be calculated. To evaluate the potential of each of the strategies, we will also perform a per protocol analysis, taking into account only those cases that were treated according to protocol. We plan a subgroup analysis based on the indication of the type of cerclage to investigate the effectiveness of the pessary compared to a primary and secondary cerclage separately, and the number of previous preterm births (overall and separately for indication for primary or secondary cerclage) in which we distinguish those with one previous preterm birth from those with two or more previous preterm births.

7.2 Secondary study parameter(s)

Time to delivery will be evaluated by Cox proportional hazard analysis and Kaplan-Meier estimates, with account for different durations of gestation at entry and stratification by type of cerclage and centre, and will be tested with the log rank test. The other secondary outcome measures will be approached similarly to the primary outcome measure. Differences in continuous outcomes between both strategies will also be assessed using a linear mixed model. Again, to account for the stratified randomization, the analysis will be adjusted for centre by fitting a random intercept for each centre and for the type of cerclage by adding the type of cerclage as a covariate to the regression model.

7.3 Other study parameters

Long Term Follow Up:

If sufficient budget becomes available the possibilities to perform a long term follow-up study will be assessed and planned. This will be added to the trial documents using an amendment. Permission to approach patients for follow-up research will be asked via the informed consent.

Cost-effectiveness analysis

For medical costs, the process of care is divided into three cost stages (antenatal stage, delivery/childbirth, postnatal stage). Health care utilization in the antenatal stage consist of admission due to threatened preterm birth, transport of patients to perinatal centres and maternal monitoring with various tests and hospital care. Costs generated during delivery are dominated by the course of childbirth and type of delivery. Resource utilization in the postnatal stage consists of maternal and neonatal health care during hospital admission (maternal ward, medium high and intensive care) and primary care following discharge. If neonatal health at discharge is suboptimal, further direct medical, direct non-medical and indirect costs may occur. Volumes of health care resource used are measured prospectively alongside the clinical study in centers as a part of the CRF. For the costs of screening we will focus on capital and operating expenses. Capital expenses include the costs of equipment. Operating expenses include the costs for disposables and personnel. For each stage and each cost category costs are measured as the volumes of resources used multiplied with appropriate valuations (unit-costs). Unit costs will be estimated according to the Dutch guideline on (unit) costing in health care. Both costs and outcomes will be discounted with 5%.

7.4 Interim analysis (if applicable)

No interim analysis for efficacy will be performed, however a first safety review is planned after all outcomes of 110 inclusions are available. This analysis will be done by an independent data and safety monitoring committee. After the first safety review, the independent data and safety monitoring committee will determine to increase or decrease the frequency of interim safety reviews. The data and safety monitoring committee can advise to stop the study for safety reasons. The following outcomes will be investigated in the interim safety review:

- Neonatal mortality
- Neonatal safety
    - Early preterm birth (< 32 weeks gestational age)
    - Duration of hospital stay for infant (stratified by pre-term vs. aterm)
    - Sepsis (early or late) or meningitis
- Maternal mortality
- Maternal safety
    - hospitalization not related to not related to delivery or threatened preterm birth

  - Damage to the cervix (cervical rupture or necrosis of the cervix)
- Pregnancy complications
  - PPROM <36 weeks gestational age
  - treated urinary or genital tract infections
  - Chorioamnionitis

Along with this a line listing of the SAE/AEs will be reported to the DSMB. Serious events that may cause concern about the safety of the study (such as maternal mortality), will be reported to the DSMB immediately if they occur.ETHICAL CONSIDERATIONS

7.5 Regulation statement

The study will be conducted according to the principles of the Declaration of Helsinki (WORLD MEDICAL ASSOCIATION DECLARATION OF HELSINKI) Ethical Principles for Medical Research Involving Human Subjects Version Edinburgh, Scotland, October 2000, with Note of Clarification on Paragraph 29 added by the WMA General Assembly, Washington 2002 end Note of Clarification on Paragraph 30 added by the WMA General Assembly, Tokyo 2004 and in accordance with the Medical Research Involving Human Subjects Act (WMO) and other guidelines, regulations and Acts.

7.6 Recruitment and consent

All women with a singleton pregnancy, history of preterm birth and a short cervical length will be informed in brief about the clinical trial by the supervising gynaecologist or by the attending resident. Subsequently, a trained research nurse will inform the patient in detail. The patient will also obtain written information about the study from the research nurse. Then the patient can think about participation in the clinical trial and discuss the study with her family. In case of participation, the informed consent form should be signed prior to randomisation.

Data on ethnicity and level of education will be collected from the patients’ medical file. The participant will give her consent for this on the consent form.

7.7 Compensation for injury

The sponsor/investigator has a liability insurance, which is in accordance with article 7, subsection 6 of the WMO.

The sponsor (also) has insurance, which is in accordance with the legal requirements in the Netherlands (Article 7 WMO and the Measure regarding Compulsory Insurance for Clinical Research in Humans of 23th June 2003). This insurance provides cover for damage to research subjects through injury or death caused by the study.

1. € 450.000,-- (i.e. four hundred and fifty thousand Euro) for death or injury for each subject who participates in the Research;
2. € 3.500.000,-- (i.e. three million five hundred thousand Euro) for death or injury for all subjects who participate in the Research;
3. € 5.000.000,-- (i.e. five million Euro) for the total damage incurred by the organisation for all damage disclosed by scientific research for the Sponsor as 'verrichter' in the meaning of said Act in each year of insurance coverage.

The insurance applies to the damage that becomes apparent during the study or within 4 years after the end of the study.

8. ADMINISTRATIVE ASPECTS, MONITORING AND PUBLICATION

8.1 Handling and storage of data and documents

Data will be collected using OpenClinica Open Source for Clinical Research, software for electronic data capture (EDC) and clinical data management (CDM). For detailed information on OpenClinica, please visit the page of OpenClinica. (https://www.openclinica.com/). The expertise for this technology is already used in the study group. Data monitoring will be done by research nurses in each of the participating centres. Data handling will be done anonymously.

8.2 Amendments

Amendments are changes made to the research after a favourable opinion by the accredited MREC has been given. All amendments will be notified to the MREC that gave a favourable opinion.
All substantial amendments will be notified to the MREC and to the competent authority.
Non-substantial amendments will not be notified to the accredited MREC and the competent authority, but will be recorded and filed by the sponsor.

8.3 Annual progress report

The sponsor/investigator will submit a summary of the progress of the trial to the accredited MREC once a year. Information will be provided on the date of inclusion of the first subject, numbers of subjects included and numbers of subjects that have completed the trial, serious adverse events/ serious adverse reactions, other problems, and amendments.

8.4 End of study report

The investigator will notify the accredited MREC of the end of the study within a period of 8 weeks. The end of the study is defined as the last patient’s last visit. In case the study is ended prematurely, the investigator will notify the accredited MREC within 15 days, including the reasons for the premature termination. Within one year after the end of the study, the investigator/sponsor will submit a final study report with the results of the study, including any publications/abstracts of the study, to the accredited MREC.

8.5 Public disclosure and publication policy

Results will be published in international and national journals. In the Netherlands the Committee on Quality Care of the NVOG will incorporate the results in the guideline on previous preterm birth. This study will be performed in at least the eight centres mentioned in this application, and at least four teaching hospitals affiliated to the eight tertiary care centres have promised to participate in the study as well.

9. STRUCTURED RISK ANALYSIS

9.1 Potential issues of concern

a. Level of knowledge about mechanism of action

Vaginal pessaries are used to prevent preterm birth since 1959 (Vitsky, 1961). During the pregnancy the cervix normally stays tightly closed with a cervical mucus plug (CMP) sealing the opening. It is hypothesized that impairment of the CMP for example by cervical effacement can lead to an ascending infection and preterm delivery; nevertheless this remains to be further clarified. The vaginal pessary encompasses the cervix and compresses the cervical canal preventing deterioration of the CMP. The pessary alternates the inclination of the cervical canal and corrects the incompetent cervix pointing forward in the axis of the vagina. It relieves direct pressure on the internal cervical os by distributing the weight of the pregnant uterus onto the vaginal floor, retrosymphyseal osteomuscular structures and Douglas cavity and may so prevent premature dilatation of the cervix and premature rupture of the membranes. Furthermore it blocks the fetal head to descend and press on the internal ostium.

b. Previous exposure of human beings with the test product(s) and/or products with a similar biological mechanism

Newcomer performed a MEDLINE search from 1966-2000 (Newcomer, 2000). This review indicated that based on non-randomized studies a pessary is potentially useful in the prevention of premature birth and seems to be without any significant risks or side effects. The only RCT of pessary prophylaxis compared a pessary to cerclage. The type of pessary used, the entry criteria and the method of randomisation, were not well described (Förster, During, & Schwarzlos, 1986). The trial recruited 112 patients in the cerclage group and 130 in the pessary group. Mean gestational age at initiation of the therapy was 27 weeks. Both methods were equal in their effects, but in view of the poor quality of the trial and the late gestational age at start of treatment, these results have little significance.

A prospective cohort study by Acharya et al. also used a double ring shaped pessary (Arabin-pessary) to treat cervical incompetence in women with a cervical length ≤ 25mm, before 30 weeks(Acharya et al., 2006). The study showed that in 55 percent of the patients, the pregnancy could be postponed to 34 weeks or more. All other reports have been of uncontrolled case series or non-randomized comparative studies.

A double ring shaped pessary is at present the most popular pessary. It has been evaluated in one cohort study (Arabin et al., 2003). This study showed in women with a twin pregnancy a mean gestational age at delivery 35+6 (33-37+4) in the pessary group (n=23) and 33+2 (24+4-37+2) in control group (n-23) (p=0.02). From the 23 women with a pessary, no one delivered before 32 weeks versus 8 women in the control group (P < .001, RR 0.12, 95% CI 0.02 to .88). Based on this study, the Arabin pessary is widely used in The Netherlands and Germany with the manufacturers selling around 2000 per year.
In April 2012 the results of the PECEP-trial were published (Goya et al., 2012). This RCT included women with singleton pregnancies who have at ultrasound examination at 18+0 to 22+6 weeks of gestation a short cervical length. Patients were randomized to a pessary or no intervention. The proportion of spontaneous deliveries before 34 weeks, the trial's primary endpoint, was reduced in women who received a cervical pessary (6% vs. 27%; odds ratio 0·18, 95% CI 0·08–0·37). These results are very impressive and promising.

g. Study population

Asymptomatic women with a singleton pregnancy and a history of preterm birth before 34 weeks of gestation will be managed according to the guideline on previous preterm birth (Otterlo, 2007). According to local protocols, and in agreement with the national guideline mentioned above, all patients will be offered the use of progesterone and cervical length measurements around 16, 18, 20 and 22 weeks gestation. In women in whom the cervix is 25 mm or less before 24 weeks gestation, the patient will be asked to participate in a randomized controlled trial.
In addition, women who are considered for a primary cerclage before 16 weeks of gestational age according to local protocols, will be eligible for the study.

j. Can effects be managed?

Treatment with the cervical cerclage or pessary can both be stopped.

9.2 Synthesis

Arabin et al. performed comparable research, showing in a questionnaire evaluation within the treatment group a general positive opinion of the treatment with the vaginal pessary (Arabin et al., 2003). 75 % of that treatment group would use the pessary again and would even recommend it to others. They

reported that there might be some increase of vaginal discharge. If there is an increase in discharge, women will be evaluated for vaginal infection and treated if necessary. Previous studies using the pessary have shown no foetal adverse effects.

10. REFERENCES

Acharya, G., Eschler, B., Grønberg, M., Hentemann, M., Ottersen, T., & Maltau, J. M. (2006). Noninvasive cerclage for the management of cervical incompetence: a prospective study. *Archives of gynecology and obstetrics*, *273*(5), 283–7. doi:10.1007/s00404-005-0082-2

Arabin, B., Halbesma, J. R., Vork, F., Hübener, M., & van Eyck, J. (2003). Is treatment with vaginal pessaries an option in patients with a sonographically detected short cervix? *J Perinat Med*, *31*(2), 122–33. doi:10.1515/JPM.2003.017

Becher, N., Adams Waldorf, K., Hein, M., & Uldbjerg, N. (2009). The cervical mucus plug: structured review of the literature. *Acta Obstet Gynecol Scand*, *88*(5), 502–13. doi:10.1080/00016340902852898

Berghella, V., Rafael, T. J., Szychowski, J. M., Rust, O. A., & Owen, J. (2011). Cerclage for short cervix on ultrasonography in women with singleton gestations and previous preterm birth: a meta-analysis. *Obstetrics and gynecology*, *117*(3), 663–71.

Esplin, M. S., O'Brien, E., Fraser, A., Kerber, R. A., Clark, E., Simonsen, S. E., … Varner, M. W. (2008). Estimating recurrence of spontaneous preterm delivery. *Obstetrics and gynecology*, *112*(3), 516–23. doi:10.1097/AOG.0b013e318184181a

Förster, F., During, R., & Schwarzlos, G. (1986). [Therapy of cervix insufficiency--cerclage or support pessary?]. *Zentralblatt für Gynäkologie*, *108*(4), 230–7.

Goya, M., Pratcorona, L., Merced, C., Rodó, C., Valle, L., Romero, A., … Carreras, E. (2012). Cervical pessary in pregnant women with a short cervix (PECEP): an open-label randomised controlled trial. *Lancet*, *379*(9828), 1800–6. doi:10.1016/S0140-6736(12)60030-0

Hein, M., Helmig, R. B., Schønheyder, H. C., Ganz, T., & Uldbjerg, N. (2001). An in vitro study of antibacterial properties of the cervical mucus plug in pregnancy. *Am J Obstet Gynecol*, *185*(3), 586–92. doi:10.1067/mob.2001.116685

Iams, J. D., Goldenberg, R. L., Mercer, B. M., Moawad, A., Thom, E., Meis, P. J., … Roberts, J. M. (1998). The Preterm Prediction Study: recurrence risk of spontaneous preterm birth. National Institute of Child Health and Human Development Maternal-Fetal Medicine Units Network. *Am J Obstet Gynecol*, *1998/06/03*(5), 1035–1040.

Liem, S., Schuit, E., Hegeman, M., Bais, J., de Boer, K., Bloemenkamp, K., … Bekedam, D. (2013). Cervical pessaries for prevention of preterm birth in women with a multiple pregnancy (ProTWIN): a multicentre, open-label randomised controlled trial. *Lancet*. doi:10.1016/S0140-6736(13)61408-7

Newcomer, J. (2000). Pessaries for the treatment of incompetent cervix and premature delivery. *Obstetrical & gynecological survey*, *55*(7), 443–8.

Otterlo, D. W. (2007). PREVENTIE RECIDIEF SPONTANE VROEGGEBOORTE.

Schaaf, J. M., Mol, B. W. J., Abu-Hanna, A., & Ravelli, A. C. J. (2011). Trends in preterm birth: singleton and multiple pregnancies in the Netherlands, 2000-2007. *BJOG*, *118*(10), 1196–204. doi:10.1111/j.1471-0528.2011.03010.x

Van der Tuuk, K., Koopmans, C. M., Groen, H., Mol, B. W., & van Pampus, M. G. (2011). Impact of the HYPITAT trial on doctors' behaviour and prevalence of eclampsia in the Netherlands. *BJOG : an international journal of obstetrics and gynaecology*, *118*(13), 1658–60. doi:10.1111/j.1471-0528.2011.03138.x

Vitsky, M. (1961). Simple treatment of the incompetent cervical os. *Am J Obstet Gynecol*, *81*, 1194–7.

Goya, M., de la Calle, M., Pratcorona, L., Merced, C., Rodó, C., Muñoz, B. & Carreras, E. (2016). Cervical pessary to prevent preterm birth in women with twin gestation and sonographic short cervix: a multicenter randomized controlled trial (PECEP-Twins). *American journal of obstetrics and gynecology*, *214*(2), 145-152.
