# Supplementary material for: Pessary or cerclage (PC study) to prevent recurrent preterm birth: a non-inferiority, randomised controlled trial
Source: eClinicalMedicine. 2024 Nov 25;78:102945. doi: 10.1016/j.eclinm.2024.102945 (PMC11626620; doi:10.1016/j.eclinm.2024.102945)
Supplement: PC Study group [file mmc3.docx]

PC Study group / PC Project Group

| **First name** | **Surname** |
| --- | --- |
| Eva | Pajkrt |
| Ben Willem | Mol |
| Brenda Miranda | Kazemier |
| Maud Désirée | Van Zijl |
| Bouchra | Koullali |
| Malou Anne | Lugthart |
| Bo Belle | Bet |
| Charlotte Elisabeth | Van Dijk |
| Annabelle Liselotte | Van Gils |
