# Supplementary material for: Pessary or cerclage (PC study) to prevent recurrent preterm birth: a non-inferiority, randomised controlled trial
Source: eClinicalMedicine. 2024 Nov 25;78:102945. doi: 10.1016/j.eclinm.2024.102945 (PMC11626620; doi:10.1016/j.eclinm.2024.102945)
Supplement: SAP_clean [file mmc4.pdf]

# Pessary or Cerclage (PC study) to prevent recurrent preterm birth – a randomized controlled trial

# -PC study-

***Statistical Analysis Plan***

| | |
|---|---|
| **EUDRA CT no.**<br><br>**Dutch Clinical trial registry no.** | <br><br>*NTR 4415, date of registration Jan 29th, 2014* |
| **Principal investigator, centre** | Prof.dr. E. Pajkrt, gynecologist |
| **Coordinating investigator** | Prof. dr. E. Pajkrt, gynecologist |
| **Sponsor** | ZonMw |
| **SAP version, date** | Version 1 |
| **Trial methodologist** | M.C. van der Weide |
| **SAP author** | A.L. van Gils |

**Names and signatures**

Please obtain dated signatures from all contributors, including the principal investigator and methodologist/statistician when a new version of the statistical analysis plan has been completed, approved by the principal investigator and other researchers and formally filed (for example in the trial master file).

| Role of contributor | Name and full affiliation | Signature | Date of signature |
|---|---|---|---|
| **Principal investigator** | E. Pajkrt, prof, PhD, MD | 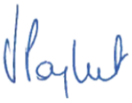 | 30 july 2023 |
| **Researcher who will perform the statistical analysis** | A.L. van Gils, MD | 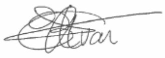 | 27 july 2023 |
| **Methodologist/statistician consulted** | M.C. van der Weide, PhD | 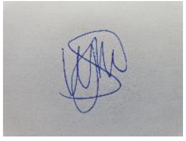 | 27 july 2023 |
| **Contributor to statistical analysis plan** | B.W. Mol, prof. PhD, MD<br>B.M. Kazemier, PhD, MD<br>C.E. van Dijk, MD | 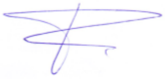<br>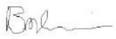<br>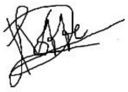 | 29 july 2023<br>29 july 2023<br>29 july 2023 |

**Revision history of statistical analysis plan**

The revision history of the statistical analysis plan includes a version number, date of approval, summary of changes, justification of revision and timing of the revisions with respect to changes to the protocol, data safety monitoring board meetings, interim analyses and the final analyses. The revision history should be formally filed (for example in the trial master file). It does not need to contain all versions made in the internal process of producing a new filed version.

| Updated statistical analysis plan version | Protocol version | Section number(s) changed | Description of and reason for changes | Date of approval |
|---|---|---|---|---|
| 1.0 | 6.0 | | | |
| | | | | |

# 1. Introduction

## 1.1 Background

Preterm birth (PTB) is defined as delivery before 37 completed weeks of gestation (GA). In the Netherlands, PTB before 37 weeks occurs in 7.7% of the pregnancies, and in 1.3% of the pregnancies even before 32 weeks (Schaaf, Mol, Abu-Hanna, & Ravelli, 2011). Consequently, it affects 12.000 pregnancies per year. PTB can occur medically indicated, e.g. when the mother suffers severe hypertensive disease or when the foetus is severely growth restricted. About 75% of the cases however occur spontaneously, sometimes after a longer episode of preterm prelabour rupture of membranes (PPROM). Preterm birth is a major contributor to perinatal mortality. Of all perinatal mortality 50-70% can be attributed to preterm birth. Similarly, spontaneous preterm birth is the leading cause of neonatal morbidity, mostly due to respiratory immaturity, intracranial haemorrhages and infections. These conditions can result in long term neurodevelopmental sequelae such as intellectual impairment, cerebral palsy, chronic lung disease, deafness and blindness. Thus, prevention of spontaneous preterm birth, with or without premature rupture of membranes, remains one of the biggest challenges in obstetric care. An important risk factor for preterm birth is previous preterm birth. Women with a history of spontaneous preterm birth have a 20% risk of recurrence of spontaneous preterm birth before 37 weeks and 15% before 34 weeks (Esplin et al., 2008; Iams et al., 1998). In the Netherlands, pregnant women with a history of preterm birth before 34 completed weeks of gestation are currently managed according to a standard protocol that has been embedded in a national guideline (Otterlo, 2007). This guideline includes the administration of intramuscular 17-hydroxy-progesterone or transvaginal progesterone from 16 gestational weeks' onwards. Moreover, cervical length is measured with transvaginal ultrasound at regular intervals between 16 and 24 weeks of GA. However, local guidelines may differ from the national guideline If a cervical length of 25 mm or less (≤25mm) is measured before 24 weeks GA, the recurrence risk of preterm birth is even more increased (Berghella et al. 2011), and this group of patients is offered a cervical cerclage. The risk of delivery before 32 weeks' gestation in the group with cerclage is 19,2% as compared to 29,5% in the group with no cerclage (RR 0,6 CI 0,48 – 0,91) (Berghella, Rafael, Szychowski, Rust, & Owen, 2011). When a cerclage is indicated, eligible women are admitted to hospital to have the cerclage surgically inserted around the cervix before 24 weeks gestation. In women with a history of multiple preterm deliveries, a primary cerclage before 16 weeks of gestational age is necessary. This procedure is performed under general or spinal anaesthesia and takes approximately 30 minutes. Women stay overnight and are discharged the next day if no complications arise. Annually approximately

100.000 multiparous women deliver in the Netherlands. Based on recent hospital numbers, we estimate that around 1% of these will have a cervical cerclage inserted, which adds up to 1.000 cerclages per year. We have recently reported that a cervical pessary is effective in the prevention of preterm birth in women with a twin pregnancy and a short cervical length (Liem et al., 2013), a finding that confirmed similar results among women with a singleton pregnancy and a short cervical length in Spain (Goya et al., 2012). Since a cervical pessary can be positioned in an outpatient setting, and costs only 40 euros, it is potentially much more attractive than the cervical cerclage. In addition, a cervical pessary is a noninvasive method contrary to a cervical cerclage which is an invasive procedure. We hypothesize that the use of a cervical pessary will be equally effective in preventing preterm birth as cervical cerclage, and therefore propose to compare the effectiveness of the two approaches in a direct randomised comparison. The outcome of the proposed study will indicate the relative effectiveness of cervical pessary for women with a singleton pregnancy and with previous preterm births and a short cervical length. Moreover, we will be able to compare the costs of both interventions. Since a pessary is much cheaper than the surgical application of a cerclage, implementation of this therapy will potentially yield a cost-reduction of 1.2 million euro per year.

### 1.2 Rationale

Preterm birth (PTB) is in quantity and in severity the most important pregnancy complication in obstetric care in the developed world. A cervical pessary and a cervical cerclage are both considered as potential preventive treatments for PTB in women with a history of preterm birth or women with a short cervical length.

### 1.3 Objective

To evaluate whether a cervical pessary can replace a cervical cerclage in women with at least one previous preterm birth and who are scheduled for cerclage, in terms of effectiveness and costs.

## 2. Endpoints

### 2.1 Primary endpoint

The primary outcome will be delivery before 32 weeks gestation.

### 2.2 Secondary endpoints

Secondary outcome measures include:

- time from intervention to delivery
- gestational age at delivery
- preterm birth rate before 24, 28, 34 and 37 weeks of gestation (overall and stratified by spontaneous or indicated)
- premature rupture of membranes
- use of tocolysis and/or corticosteroids during pregnancy
- mode of delivery
- maternal infections
- maternal side effects (i.e. vaginal discharge, bleeding, discomfort)
- neonatal and maternal admissions

Perinatal outcome will be assessed through a composite of adverse perinatal outcome. For this secondary outcome to be true, one or more of the following composite outcomes need to be found:

- Chronic lung disease
- Intraventricular Haemorrhage (IVH) grade III and IV: defined as hemorrhage in the germinal matrix, ventricles, or cerebral parenchyma; observed on ultrasound examination or MRI. Ultrasound examination is routinely performed in all neonates born prior to 32 weeks GA, or in neonates with neurologic symptoms.
- Periventricular Leucomalacia (PVL) higher then grade I: Periventricular lucency in the white matter
- Necrotizing Enterocolitis (NEC) higher then stage I: defined as the presence of the characteristic clinical features of abdominal distention, with or without rectal bleeding, and abdominal radiographic finding associated with pneumatosis intestinalis (this last finding is an abnormal gas pattern with dilated loops consistent with ileus)
- Retinopathy of Prematurity (ROP)
- Patent ductus arteriosus (PDA)

- Treated seizures: defined as convulsions that had to be treated (yes / no)
- Early and late sepsis: classified as suspected or proven (caused by any pathogen) and defined as a neonatal infection with cardiorespiratory instability or a positive blood culture caused by any pathogen. Clinical infection will include symptoms like positive findings on clinical exam, imaging or laboratory tests. Laboratory signs of infection will be increased CRP, leukocytosis or leukocytopenia.
- Neonatal meningitis: classified as suspected or proven (caused by any pathogen). Clinical infection will include symptoms like positive findings on clinical exam, imaging, or laboratory tests. Laboratory signs of infection will be increased CRP, leukocytosis or leukocytopenia.
- (Intrapartum) stillbirth
- Death before discharge from the hospital

# 3. Study methods

## 3.1 Study design

This is a non-inferiority, multicentre randomized controlled trial that will compare the effectiveness of a cervical pessary to the standard treatment (cervical cerclage) in women with a singleton pregnancy and an indication for a primary or secondary cerclage.

## 3.2 Study population

Asymptomatic women with a singleton pregnancy and a history of preterm birth before 34 weeks' gestation and women with a history of multiple preterm births will be managed according to the guideline on previous preterm birth (Otterlo, 2007). All patients will be treated according to local protocols. Most patients will be offered the use of progesterone and cervical length measurements around 16, 18, 20 and 22 weeks' gestation, however the exact treatment protocol could vary between different hospitals. In women in whom the cervix is 25 mm or shorter before 24 weeks' gestation, the patient will be asked to participate in a randomized controlled trial. Women who are considered for a primary intervention before 16 weeks gestation, will also be asked to participate in this trial.

## 3.3 Inclusion criteria

In order to be eligible to participate in this study, a subject must meet all of the following criteria:

1. Singleton pregnancy with a history of spontaneous preterm birth before 34 weeks of gestation
2. Cervical length of 25mm or less on transvaginal ultrasound before 24 weeks of GA OR Indication for primary cerclage before 16 weeks in current pregnancy based on obstetric history, according to local protocol
3. Written informed consent

## 3.4 Exclusion criteria

A potential subject who meets any of the following criteria will be excluded from participation in this study:

1. Maternal age less than 18 years
2. Inability to give informed consent
3. Placenta praevia
4. Vasa praevia

5. Premature Prelabour Rupture of the Membranes (PPROM)

6. Cervical dilatation ≥3cm

7. Cervical length <2mm

8. Identified major congenital abnormalities: Major fetal abnormalities are defined as those that are lethal or require intensive prenatal care or postnatal surgery.

9. Women with clinical signs of chorioamnionitis or signs of intra uterine infection, defined as a temperature > 37,8 degrees Celsius, maternal tachycardia > 100 beats per minute and fetal tachycardia > 160 beats per minute, without any other focus of infection

### 3.5 Treatment of subjects

Eligible women will be randomly allocated to receive either a cervical cerclage or a cervical pessary. Both will be placed in situ before 24 weeks, or before 16 weeks in case of a primary intervention, and will stay in situ up to 36 weeks gestation or until delivery, whatever comes first. In all women, we will measure cervical length prior to randomisation except in those who are considered for primary intervention before 16 weeks of gestation. Transvaginal ultrasonography will be carried out with a 5-MHz transducer. The probe will be placed in the anterior fornix of the vagina and a sagittal view of the cervix, with the echogenic endocervical mucosa along the length of the canal, will be obtained. The callipers will be used to measure the distance between the triangular area of echodensity at the external os and the V-shaped notch at the internal os. The presence or absence of funnelling at the internal os will also be recorded. All women participating will be enrolled after transvaginal measurement for cervical length before 24 weeks or before 16 weeks when primary intervention is required. Next to this research intervention cases are treated according to the local protocol in the participating clinics and other interventions i.e. tocolysis and corticosteroids in case of a threatened preterm birth can be carried out as usual. Furthermore, no extra interventions will be needed. The pessary is made of silicone which is soft and flexible. It is folded and put around the cervix by a simple vaginal examination without causing any pain. In case of complaints, examination of the patient is advised to reposition the pessary or to replace the pessary with another size if necessary. The cervical cerclage will be placed under general or spinal anesthesia.

### 3.6 Blinding

Due to the type of interventions, this study will not be blinded.

### 3.7 Randomisation procedure

GCP trained nurses will counsel patients, ask informed consent, perform randomization and collect data. Randomization will be centrally controlled using an on-line computerized randomisation service, once patient data have been entered in a web-based database. Randomization will be centrally controlled using an 24hr/7day accessible on-line computerized randomization service ( *ALEA Clinical software version 16 (FormsVision, Abcoude, The Netherlands) and from July 2022 in Castor Electronic Data Capture v2022.3.2.0).* Subjects will be randomized in a 1:1 ratio to cervical cerclage and pessary. Randomization will be stratified by indication for type of cerclage (primary or secondary) and centre (to prevent any imbalance between groups in aspects of maternal or neonatal care that may differ between centres). We will apply block randomisation with a variable block size of 2 and 4.

## 4. Sample-size

We plan to evaluate the non-inferiority of a cervical pessary as compared to cervical cerclage. We assume an event rate of 20% for the primary outcome, i.e. delivery before 32 weeks, for cerclage based on current literature. We will use a non-inferiority margin of 10%. This is equivalent to saying that pessarium is non-inferior to cerclage when the upper limit of the 95% confidence interval of the event rate of the primary outcome in the pessarium group is less than 30%. Using a one-sided alpha of 0.05 and power of 0.80 we need 2 groups of 200 women.

# 5. Analysis considerations

## 5.1 Analysis populations

### 5.1.1 Full analysis population (ITT)

Data will initially be analyzed according the intention to treat principle. It includes all randomized patients who gave a signed informed consent for the study, regardless of protocol deviations or additional therapies, like a cervical cerclage. In the intention to treat analysis, patients will be analysed in groups according to the allocated treatment at randomisation. Patients who withdraw from the study will remain in their treatment group for the final analysis according to the intention-to-treat principle. Every effort will be made to obtain complete information on each patient randomized. The only reason for not obtaining complete information is that the patient was lost to follow up or that she withdraws consent to access her medical chart after delivery. Randomised women who appear to fail inclusion and exclusion criteria (eligibility violations) during blinded data review, will be excluded from this analysis. This will only be done for criteria that were present at the time of randomisation. Two clinicians will review such cases and where there are discrepancies, a third will be consulted. The following protocol violations will be considered:

1. Maternal age less than 18 years
2. Inability to give informed consent
3. Placenta praevia
4. Vasa praevia
5. Premature Prelabour Rupture of the Membranes (PPROM)
6. Cervical dilatation ≥3cm
7. Cervical length <2mm
8. Identified major congenital abnormalities: Major fetal abnormalities are defined as those that are lethal or require intensive prenatal care or postnatal surgery.
9. Women with clinical signs of chorioamnionitis or signs of intra uterine infection, defined as a temperature > 37,8 degrees Celsius, maternal tachycardia > 100 beats per minute and fetal tachycardia > 160 beats per minute, without any other focus of infection

### 5.1.2 Per Protocol population

To evaluate the potential of each of the strategies, we will also perform a per protocol analysis, taking into account only those cases that were treated according to protocol, which is:

- A patient meets all inclusion and none of the exclusion criteria.
- The randomized treatment has been continued preferably up to 36 weeks of gestation or until (threatened) preterm delivery, whichever came first.
- In case of removal of intervention before 36 weeks of gestation or delivery:
    - o In case of cerclage: the cerclage was in situ at least 80% of the possible days the cerclage could have been in situ.
    - o In case of a pessary, the device must have been in place at least 80% of the days it was possible to have it inserted.

### 5.2 Covariates and Subgroups

The primary outcome (rate of PTB < 32 weeks, (n/N, %)) for the following subgroup analyses are pre-specified in the protocol:

- The indication for intervention: primary (obstetric history indicated) vs secondary (ultra-sound guided indicated
- The number of previous preterm births in which we distinguish those with one previous preterm birth from those with two or more previous preterm births. (1 previous PTB vs. ≥2 previous PTB)
- Cervical length ≤15mm vs >15mm within the group of participants with a secondary (ultrasound guided) indication for an intervention.
- Funneling yes / no within the group of participants with a secondary (ultrasound guided) indication for an intervention.

Subgroup analysis will be performed by including an interaction term with the treatment allocation (pessary vs. cerclage). When the interaction will be found statistically significant ($p<0.05$) we will estimate the treatment effect within the different strata of the subgroup.

### 5.3 Missing Data

When data of the delivery and follow-up of the neonate cannot be obtained, the patient will be categorized as lost to follow up and not be included in both intention to treat and the per protocol analyses. In all other cases, the patient will be included in the intention to treat analyses and depending on the missing values in the per protocol analyses.

## 5.4 Interim Analyses and Data Monitoring

No interim analysis for efficacy will be performed, however a first safety review is planned after all outcomes of 110 inclusions are available. This analysis will be done by an independent data and safety monitoring committee. After the first safety review, the independent data and safety monitoring committee will determine to increase or decrease the frequency of interim safety reviews. The data and safety monitoring committee can advise to stop the study for safety reasons. The following outcomes will be investigated in the interim safety review:

- Neonatal mortality
- Neonatal safety
    - Early preterm birth (< 32 weeks gestational age)
    - Duration of hospital stay for infant (stratified by pre-term vs. aterm)
    - Sepsis (early or late) or meningitis
- Maternal mortality
- Maternal safety
- hospitalization not related to not related to delivery or threatened preterm birth.
- Damage to the cervix (cervical rupture or necrosis of the cervix)
- Pregnancy complications
    - PPROM
    - Treated urinary or genital tract infections
    - Chorioamnionitis

Along with this a line listing of the SAE/AEs will be reported to the DSMB. Serious events that may cause concern about the safety of the study (such as maternal mortality), will be reported to the DSMB immediately if they occur.

# 6. Efficacy analyses

## 6.1 Timing of final statistical analysis

The statistical analysis for the primary outcome (PTB < 32 weeks ) and preterm birth rate before 24, 28, 32, 34 and 37 weeks (spontaneous, iatrogenic and total) can be performed after 10 weeks of the delivery of the last child.

After data cleaning for all outcomes has been completed, the primary outcome and all secondary outcomes can be analysed. It is expected that this will occur within four to six months of the birth of the last child.

## 6.2 Primary endpoint analysis

Data will initially be analyzed according the intention to treat method. We will assess the differences between the two groups by calculating the relative risk of the main outcome with a log-binomial regression model. Stratification by centre and type of indication for intervention (primary or secondary) will be adjusted for with centre and indication as covariates. For the primary outcome, also the absolute risk difference will be presented. Using the Farrington-Manning test, the non-inferiority hypothesis will be tested using the predetermined non-inferiority margin of 10% (risk difference). When appropriate, numbers needed to treat will be calculated. To evaluate the potential of each of the strategies, we will also perform a per protocol analysis, taking into account only those cases that were treated according to protocol.

## 6.3 Secondary efficacy analyses

- For dichotomous outcomes, generalized linear regression (glm) analysis using a log link will be performed to calculate Relative Risks. The 95% confidence intervals and the p-value will also be presented. When there are ≤5 events for a variable, Fisher's exact test will be used to calculate the p-value instead of the glm model.
- The distribution of continuous outcomes will be inspected visually. For approximately normally distributed continuous outcomes, means and standard deviations will be reported and difference in means will be calculated. Mean differences and the corresponding 95% confidence intervals will be presented along with the p-value from the t-test. For highly skewed continuous outcomes, medians and interquartile ranges will be reported together with the differences in medians.

- Time to delivery will be evaluated by Cox proportional hazard analysis and Kaplan-Meier estimates, with account for different durations of gestation at entry and stratification by type of cerclage and centre, and will be tested with the log rank test.
- Besides, we will explore the PTB rates (spontaneous and total) within primary and secondary intervention groups.
- All statistical tests will use a 2-sided p-value of 0.05. All confidence intervals presented will be 95% and two-sided. There will be no adjustment of p-values, as no interim analysis was performed.

Time to delivery will be evaluated by Cox proportional hazard analysis and Kaplan-Meier estimates, with account for different durations of gestation at entry and stratification by type of cerclage and centre, and will be tested with the log rank test. The other secondary outcome measures will be approached similarly to the primary outcome measure. Differences in continuous outcomes between both strategies will also be assessed using a linear mixed model. Again, to account for the stratified randomization, the analysis will be adjusted for centre by fitting a random intercept for each centre and for the type of cerclage by adding the type of cerclage as a covariate to the regression model.

## 7. Safety analyses

All serious adverse events (SAE's) occurring during the study will be listed individually in the supplementary information. Listings will be divided according to treatment group. All serious adverse events that are considered to be possibly related to the study medication by the investigators will be marked.

### 7.1 Deaths, Serious Adverse Events and other Significant Adverse Events

A serious adverse event (SAE) is any untoward medical occurrence or effect that at any dose:

- Results in maternal death;
- Is life threatening (at the time of the event) to the mother;
- Requires hospitalization or prolongation of existing inpatients' hospitalization other than expected obstetric complications (such as threatened premature labour, admissions due to labour or scheduled delivery/ caesarean section);
- Results in persistent or significant disability or incapacity of the mother;
- Is a severe congenital anomaly or birth defect of the neonate; or

- Any other important medical event that may not result in death, be life threatening, or require hospitalization, may be considered a serious adverse experience when, based upon appropriate medical judgement, the event may jeopardize the subject or may require an intervention to prevent one of the outcomes listed above.

## 8. Comparison to study protocol

In the protocol a cost-effectiveness analysis was planned, but we will not conduct that analysis now. Therefore, we adjusted the secondary outcomes and removed all measures required for cost-effectiveness treatment, namely use of tocolysis, corticosteroids, magnesium-sulphate and maternal admission days.

Dyspareunia was removed as a secondary outcome for it was not stated in the CRF and consequently no data was collected on this specific item.

One additional subgroup analyses was added within the group of participants with a secondary indication for an intervention based on short cervical length ≤25 mm, namely funneling yes/no.

To adjust for stratification in the randomization procedure, instead of using a random intercept, stratification by centre and indication will be accounted for in the primary analysis by adding these factors as covariates in the regression model, as advised in the guideline on adjustment of baseline covariates in clinical trials by the EMA.

The Farrington-Manning test was added to test the non-inferiority assumption.

# 9. Presentation of study results

## 9.1 Recruitment

The recruitment of study participants will be presented using the CONSORT flow diagram. Exact details of screened patients cannot be given, since cervical length screening is standardized care for patients with sPTB < 34 weeks and no information is available on the number of women who received these routine cervical length screening. .

## 9.2 Protocol violations

Severe protocol violations will be pointed out in the Consort Flow figure with reason of violation.

## 9.3 Baseline characterisations

The baseline characteristics will be presented for the total population as randomised (intention-to-treat), using the format of the mock table included below. Data will be presented using absolute numbers with percentages for discrete outcomes. Continuous outcomes will be presented as means with standard deviation, or medians with interquartile ranges.

| | Pessary (n = ) | Cerclage (n = ) |
|---|---|---|
| **Maternal characteristics** | | |
| Maternal age, years (mean, SD) | XX (xx) | XX (xx) |
| Body-mass index, kg/m$^2$ (mean, SD) | XX.X (xx) | XX.X (xx) |
| Education<br>Low* | NNN (%) | NNN (%) |
| Middle and high** | NNN (%) | NNN (%) |
| Ethnicity | | |
| White | NNN (%) | NNN (%) |
| Black | NNN (%) | NNN (%) |
| Middle Eastern | NNN (%) | NNN (%) |
| Asian | NNN (%) | NNN (%) |
| Other | NNN (%) | NNN (%) |
| Unknown | NNN (%) | NNN (%) |
| Other origins | NNN (%) | NNN (%) |
| Current smoker | NNN (%) | NNN (%) |
| History of cervical surgery (Conisation/LLETZ***) | NNN (%) | NNN (%) |
| History of uterine surgery | NNN (%) | NNN (%) |
| Smoking | NNN (%) | NNN (%) |
| Recurrent urinary tract infections (>3 per year) | NNN (%) | NNN (%) |
| Uterus anomaly | NNN (%) | NNN (%) |
| **Obstetric characteristics** | | |
| Indication for intervention | | |
| Primary indication <16 weeks | NNN (%) | NNN (%) |
| Secondary indication cervical length < 25mm | NNN (%) | NNN (%) |
| Number of previous preterm birth $<34^{+0}$ | NNN (%) | NNN (%) |

| | | |
|---|---|---|
| History of curettage | NNN (%) | NNN (%) |
| Conception | | |
| Pregnancy after IVF/ ICSI**** | NNN (%) | NNN (%) |
| Nugent score in first trimester | | |
| <4 | NNN (%) | NNN (%) |
| 4-7 | NNN (%) | NNN (%) |
| >7 | NNN (%) | NNN (%) |
| Not performed | NNN (%) | NNN (%) |
| Unknown | NNN (%) | NNN (%) |
| Vaginal swab | | |
| Negative | NNN (%) | NNN (%) |
| GBS | NNN (%) | NNN (%) |
| E coli | NNN (%) | NNN (%) |
| Trichomonas | NNN (%) | NNN (%) |
| Candida | NNN (%) | NNN (%) |
| Other | NNN (%) | NNN (%) |
| Not performed | NNN (%) | NNN (%) |
| Unknown | NNN (%) | NNN (%) |
| Gestational age (w+d) at randomization (median, IQR) | XX+X ( XX+X - XX+X) | XX+X ( XX+X - XX+X) |
| Cervical length at randomization (mm) (mean, SD) | XX.X (xx) | XX.X (xx) |
| Cervical length range | NNN (%) | NNN (%) |
| 0-15 mm | NNN (%) | NNN (%) |
| 16-25 mm | NNN (%) | NNN (%) |
| **>25mm (primary indication only)** | | |
| Funneling | NNN (%) | NNN (%) |

* Primary school, prevocational secondary education (VMBO in Dutch)

** Senior general secondary education (HAVO in Dutch), pre-university secondary education (VWO in Dutch), secondar vocational education (MBO in Dutch), higher professional education (HBO in Dutch), and university education (WO in Dutch).

***LLETZ, large loop excision of the transformation zone

****ISCI, intracytoplasmic sperm injections, IVF, in vitro fertilization

**Table 1: Baseline characteristics**

## 9.4 Primary and secondary outcomes

The primary and secondary outcomes be presented for the total population as randomised (intention-to-treat) using the format of the mock table included below.

| | Pessary | Cerclage | RR (95% CI) | RD (95% CI) | p-value |
|---|---|---|---|---|---|
| Primary outcome | | | | | |
| PTB < 32 weeks (ITT) crude | NNN (%) | NNN (%) | RR (95% CI) | | 0.XX |
| PTB < 32 weeks (ITT) adjusted | NNN (%) | NNN (%) | RR (95% CI) | | 0.XX |
| PTB < 32 weeks (PP) crude | NNN (%) | NNN (%) | RR (95% CI) | | 0.XX |
| PTB < 32 weeks (PP) adjusted | NNN (%) | NNN (%) | RR (95% CI) | | 0.XX |
| Obstetric outcomes | | | | | |
| PTB < 37 weeks | NNN (%) | NNN (%) | RR (95% CI) | | 0.XX |
| sPTB < 37 weeks | NNN (%) | NNN (%) | RR (95% CI) | | 0.XX |
| PTB < 34 weeks | NNN (%) | NNN (%) | RR (95% CI) | | 0.XX |
| sPTB < 34 weeks | NNN (%) | NNN (%) | RR (95% CI) | | 0.XX |
| PTB < 32 weeks | NNN (%) | NNN (%) | RR (95% CI) | | 0.XX |
| sPTB < 32 weeks | NNN (%) | NNN (%) | RR (95% CI) | | 0.XX |
| PTB < 28 weeks | NNN (%) | NNN (%) | RR (95% CI) | | 0.XX |
| sPTB < 28 weeks | NNN (%) | NNN (%) | RR (95% CI) | | 0.XX |
| PTB < 24 weeks | NNN (%) | NNN (%) | RR (95% CI) | | 0.XX |
| sPTB < 24 weeks | NNN (%) | NNN (%) | RR (95% CI) | | 0.XX |
| Time to delivery (days), mean (SD) | XX.X (xx) | XX.X (xx) | | | 0.XX |
| Gestational age at delivery | | | | | |
| Use of progesterone | NNN (%) | NNN (%) | RR (95% CI) | | 0.XX |
| PPROM^ | NNN (%) | NNN (%) | RR (95% CI) | | 0.XX |

| | | | | |
|---|---|---|---|---|
| Mode of delivery | | | | |
| Vaginally | NNN (%) | NNN (%) | RR (95% CI) | 0.XX |
| Caesarean section | NNN (%) | NNN (%) | RR (95% CI) | 0.XX |
| Use of | | | | |
| Tocolytics | NNN (%) | NNN (%) | RR (95% CI) | 0.XX |
| Corticosteroids | NNN (%) | NNN (%) | RR (95% CI) | 0.XX |
| Neonatal outcomes | | | | |
| Composite adverse neonatal outcome (ITT), crude | NNN (%) | NNN (%) | RR (95% CI) | 0.XX |
| Chronic Lung disease* | NNN (%) | NNN (%) | RR (95% CI) | 0.XX |
| IVH grade III or IV* | NNN (%) | NNN (%) | RR (95% CI) | 0.XX |
| PVL > grade 1* | NNN (%) | NNN (%) | RR (95% CI) | 0.XX |
| NEC > stage 1* | NNN (%) | NNN (%) | RR (95% CI) | 0.XX |
| ROP* | NNN (%) | NNN (%) | RR (95% CI) | 0.XX |
| Patent ductus arteriosus | NNN (%) | NNN (%) | RR (95% CI) | 0.XX |
| Treated seizures | NNN (%) | NNN (%) | RR (95% CI) | 0.XX |
| Culture proven sepsis | | | | |
| < 72 hours after birth (early) | NNN (%) | NNN (%) | RR (95% CI) | 0.XX |
| > 72 hours after birth (late) | NNN (%) | NNN (%) | RR (95% CI) | 0.XX |
| Meningitis | NNN (%) | NNN (%) | RR (95% CI) | 0.XX |
| Perinatal death | NNN (%) | NNN (%) | RR (95% CI) | 0.XX |
| Stillbirth < 24 weeks | NNN (%) | NNN (%) | RR (95% CI) | 0.XX |
| Neonatal death > 24 weeks | NNN (%) | NNN (%) | RR (95% CI) | 0.XX |
| Maternal outcomes | | | | |
| Maternal mortality | NNN (%) | NNN (%) | RR (95% CI) | 0.XX |
| Maternal morbidity | | | | |
| Infections | NNN (%) | NNN (%) | RR (95% CI) | 0.XX |
| Treated genital tract infections | NNN (%) | NNN (%) | RR (95% CI) | 0.XX |
| Treated urinary tract infections | NNN (%) | NNN (%) | RR (95% CI) | 0.XX |
| Chorioamnionitis | NNN (%) | NNN (%) | RR (95% CI) | 0.XX |
| Maternal side effects | | | | |
| Vaginal discharge | NNN (%) | NNN (%) | RR (95% CI) | 0.XX |
| Vaginal blood loss | NNN (%) | NNN (%) | RR (95% CI) | 0.XX |
| Discomfort or pain | NNN (%) | NNN (%) | RR (95% CI) | 0.XX |

**^PPROM: premature rupture of membranes**
*** Severe respiratory distress syndrome (RDS) or Bronchopulmonary Dysplasia (BPD), NEC: Necrotizing Enterocolitis, PVL: Periventricular leukomalacia, ROP: Retinopathy of prematurity, IVH: Intraventricular Hemorrhage**

*Table 2– Primary and secondary outcomes*

## 9.5. Subgroup analyses

| | Pessary | Cerclage | Relative Risk (95% CI) | p-value interaction term |
|---|---|---|---|---|
| **PTB < 32 weeks (primary outcome)** | | | | |
| Primary indication | N/ NN (%) | N/ NN (%) | | |
| Secondary indication | N/ NN (%) | N/ NN (%) | | |
| | | | | |
| 1 previous PTB | N/ NN (%) | N/ NN (%) | | |
| > 1 previous PTB | N/ NN (%) | N/ NN (%) | | |
| Within ultrasound guided intervention group | | | | |
| Cervical length < 15mm | N/ NN (%) | N/ NN (%) | | |
| Cervical length 15-25mm | N/ NN (%) | N/ NN (%) | | |
| | | | | |
| Funneling yes | N/ NN (%) | N/ NN (%) | | |
| Funneling no | N/ NN (%) | N/ NN (%) | | |

***Table 3– Subgroup analyses on the primary outcome PTB < 32 weeks***

| | Pessary<br>N= NN | Cerclage<br>N=NN |
|---|---|---|
| **PRIMARY INDICATION** | | |
| PTB < 37 weeks | NN (%) | NN (%) |
| sPTB < 37 weeks | NN (%) | NN (%) |
| PTB < 34 weeks | NN (%) | NN (%) |
| sPTB < 34 weeks | NN (%) | NN (%) |
| PTB < 32 weeks | NN (%) | NN (%) |
| sPTB < 32 weeks | NN (%) | NN (%) |
| PTB < 28 weeks | NN (%) | NN (%) |
| sPTB < 28 weeks | NN (%) | NN (%) |
| PTB < 24 weeks | NN (%) | NN (%) |
| sPTB < 24 weeks | NN (%) | NN (%) |
| **SECONDARY INDICATION** | | |
| PTB < 37 weeks | NN (%) | NN (%) |
| sPTB < 37 weeks | NN (%) | NN (%) |
| PTB < 34 weeks | NN (%) | NN (%) |
| sPTB < 34 weeks | NN (%) | NN (%) |
| PTB < 32 weeks | NN (%) | NN (%) |
| sPTB < 32 weeks | NN (%) | NN (%) |
| PTB < 28 weeks | NN (%) | NN (%) |
| sPTB < 28 weeks | NN (%) | NN (%) |
| PTB < 24 weeks | NN (%) | NN (%) |
| sPTB < 24 weeks | NN (%) | NN (%) |

**Table 4– Exploratory analysis on (s)PTB rates <24, < 28, <32, <34 and <37 weeks per primary or secondary indication**

### 9.5 Additional tables for supplementary

| Serious Adverse Events | Pessary *(n=NNN)* | Cerclage *(n=NNN)* | Relative Risk (95% CI) | p-value subgroup |
|---|---|---|---|---|
| Maternal death | NNN (%) | NNN (%) | RR (95% CI) | 0.XX |
| Life threatening (at the time of event) to the mother | NNN (%) | NNN (%) | RR (95% CI) | 0.XX |
| Hospitalization or prolongation for other than expected reason# | NNN (%) | NNN (%) | RR (95% CI) | 0.XX |
| Persistent or significant disability or incapacity of the mother | NNN (%) | NNN (%) | RR (95% CI) | 0.XX |
| Severe congenital anomaly or birth defect of the neonate | NNN (%) | NNN (%) | RR (95% CI) | 0.XX |
| Any other important medical event$ | NNN (%) | NNN (%) | RR (95% CI) | 0.XX |

#Requires hospitalization or prolongation of existing inpatients' hospitalization other than expected obstetric complications (such as threatened premature labour, admissions due to labour or scheduled
$Any other important medical event that may not result in death, be life threatening, or require hospitalization, may be considered a serious adverse experience when, based upon appropriate medical judgement, the event may jeopardize the subject or may require an intervention to prevent one of the outcomes listed above.

**Table 1– Serious Adverse Events**

| | **Pessary (n = )** | **Cerclage (n = )** |
|---|---|---|
| Allocated treatment initiated | NNN (%) | NNN (%) |
| Removal of cerclage or pessary | | |
| According to study protocol | | |
| Gestational age > 36 weeks | NNN (%) | NNN (%) |
| Contractions or labour | NNN (%) | NNN (%) |
| Other reasons, required for delivery | NNN (%) | NNN (%) |
| (P)PROM | NNN (%) | NNN (%) |
| Not according to study protocol | | |
| Discomfort | NNN (%) | NNN (%) |
| Excessive discharge | NNN (%) | NNN (%) |
| Patient preference | NNN (%) | NNN (%) |
| Pessary fell out | NNN (%) | NNN (%) |
| Vaginal blood loss | NNN (%) | NNN (%) |
| Replacement of pessary or cerclage* | | |

*If reason for removal other than GA >36 weeks, (P)PROM, contractions/labour, delivery

**Table 2 - Details on use of intervention**

## 10. Figures

### 10.1 Flowchart of participants

The flow of study participants will be presented using the CONSORT flow-chart for clinical trial participants as shown below.

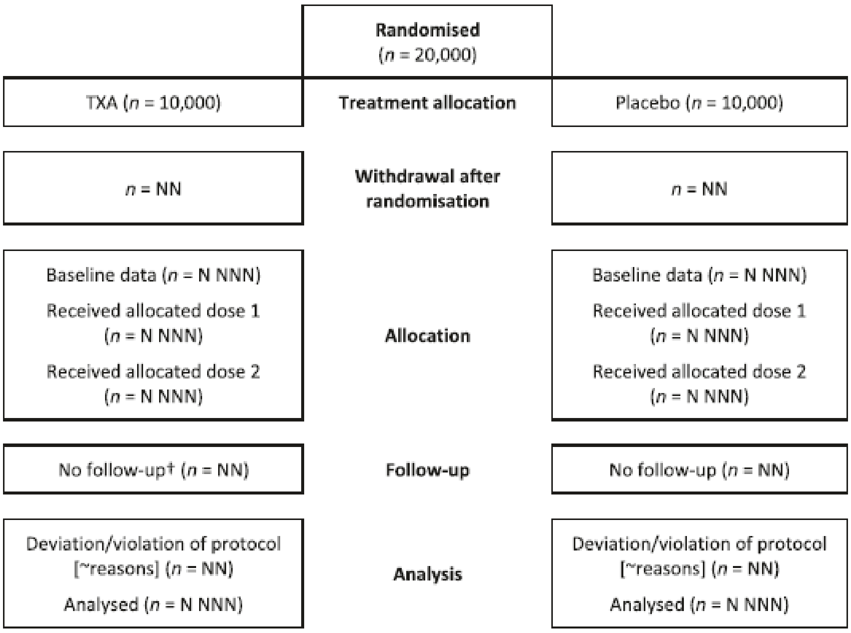


**Fig. 1** Trial profile. †No follow-up relates to those patients where there is no information on the primary endpoint

### 10.2 Kaplan Meier curves for time to delivery

Kaplan-Meier plots will be constructed to illustrate the time to delivery from the day of randomization to delivery for the intention-to-treat population, as well as for the subgroups per indication. The difference in gestational age at birth between the treatment groups will be tested using a log-rank test.
